# Supplementary material for: The Google matrix controls the stability of structured ecological and biological networks
Source: Nat Commun. 2016 Sep 30;7:12857. doi: 10.1038/ncomms12857 (PMC5056432; doi:10.1038/ncomms12857)
Supplement: Supplementary Information — Supplementary Figures 1-24, Supplementary Notes 1-4 and Supplementary References [file ncomms12857-s1.pdf]

Supplementary Note 1: Competition Model

1A.The Lotka-Volterra Model, Scaling and Ensemble Model

If we let N_i be the abundance of the i'th species, the Lotka-Volterra equations for a total of *n*-species can be written in the usual form:

$$\frac{dN_i}{dt} = \frac{R_i}{k_i} N_i (k_i - \sum_j \alpha_{ij} N_j) \quad i=1,2,..., n \tag{1}$$

Here R_i is the birth-rate of the i'th species k_i its carrying capacity, and α_{ij} the interaction coefficient representing the effect species-j has on species-i. The model may be reparameterised by taking $a_{ij} = \frac{\alpha_{ij}}{k_i}$, $r_i = \frac{R_i}{k_i}$,

so that (1) becomes: $$\frac{dN_i}{dt} = r_i N_i (1 - \sum_j a_{ij} N_j) \tag{2}$$

We use here one of the more popular scalings (see eg., Gilpin and Case (1976, 1981), Pomerantz and Gilpin (1979); Roberts (1974, 1984, 1989); Kokkoris & Janssen (2002); Janssen & Kokkoris (2003), Stone & Roberts (1991); Roberts & Stone (2004)), and up to a scaling factor is identical to the competition module used in Rohr et al. (2015) -- referred to here as RSB. Owing to the vast number of systems described by (2) the equations have been normalized as recommended by taking all species to be self-regulated with a_{ii}= 1 and with carrying capacities set to unity. This in effect scales to unity each equilibrium population in the absence of other species. The key results that follow hold for other or relaxed versions of these scalings (see SN1-K below).

The Ensemble Model: Interspecific interactions are taken to be of the form

$$a_{ij} = c + b_{ij} \quad . \tag{3}$$

Here the b_{ij} are random perturbations having mean zero and variance Var(b_{ij})=σ^2. The b_{ij} are selected randomly (uniformly) from the interval $[-cv, +cv]$ with spread $0 \leq v \leq 1$, so that $< b_{ij} > = 0$ and $Var(b_{ij}) = \frac{c^2 v^2}{3} = \sigma^2$. This ensures a_{ij} >0 thereby ensuring systems of pure competition.

An ensemble of *n*-species competition can be constructed in which every pairwise interaction term a_{ij} has mean strength term <a_{ij}>=–c and variance Var(b_{ij})= $\mathrm{Var}(a_{ij}) = \sigma^2$. The ensemble can be specified completely by the parameters (m, c, σ); however it is more convenient to specify the ensemble by the triple (*n*, c, γ) where γ is defined as:

$$\gamma = \sqrt{n-1}\, \sigma/(1-c) \; . \tag{4}$$

Each interaction matrix in the ensemble represents a distinct ecological system, having its own unique dynamics and density-dependence relationships. The ensemble is considered to represent the totality of possible interaction matrices a particular competition community might possess over its complete lifetime given that it is undergoing structural disturbances of mean strength represented by γ. Each matrix has its own equilibrium solution $N=N^*$. Hence the ensemble of matrices gives rise to a corresponding ensemble of equilibrium points which could be envisaged in the form of a "stochastic cloud." Thus a study of the equilibrium points yields information regarding the likelihood of community coexistence.

In this model, environmental fluctuations make the interaction strengths vary about the community's mean strength of competition. Thus two communities may both have the same average interaction strength <a_{ij}>= –c, but the one undergoing stronger perturbation will show a greater variation in its interaction

coefficients. Hence the stochastic model associates increasing disturbance with an increase in $\mathrm{Var}(a_{ij}) = \sigma^2$(the variance of the perturbation b_{ij}) and thus γ. It is thus convenient to represent the level of disturbance in the community by γ.

1B. Approximating population equilibria N_i^* of competition ensemble model

The system has equilibrium populations which are solutions of the AN^*=1. As shown in Stone (1988) earranging this gives:

$$[(1-c)I + B]N^* = (1-c\mathrm{T})e \quad where\ \ \mathrm{T} = \textstyle\sum_{i=1}^{n} N_i^*, \text{ or} \tag{5}$$

$$N^* = \kappa[I + B']^{-1}e \qquad \text{where} \quad \kappa = 1\text{-}cT$$

and the symbol $'$ represents a division by (1-c). Assuming $\rho(B') < 1$, we can expand the inverse matrix to obtain the first order approximation:

$$N_i^* \approx \kappa\left(1 - \textstyle\sum_{j=1}^{n} b'_{ij}\right) \tag{6}$$

Let $\gamma = \frac{\sqrt{n-1}\,\sigma}{1-c}$. By May-Wigner theorem the spectral radius of the matrix B' is $\rho(B') = \gamma$ (section SN1-G below and May *(1)*). Thus the approximation above requires the condition $\gamma < 1$.

On positivity of term *κ=1-cT>0:* Examining Eq.1, we see that *κ=1-cT* is an eigenvalue of matrix $S^\dagger = D[(1-c)I + B]$. Thus a sufficient condition for positive κ is that the stability matrix $S^\dagger$ has all eigenvalues positive, or that $S^\dagger$ is locally stable. Certainly $\kappa < 0$ implies that $S^\dagger$ is unstable

In Section SN1-I we show that $S^\dagger$ is locally stable for $\gamma < 1$ and thus 1-cT>0 in large feasible systems.

Additional note: Consider again the approximation $N_i^* \approx (1-cT)\left(1 - \sum_{j=1}^{n} b'_{ij}\right)$. We are interested in the first species that goes extinct, or the point when feasibility is first lost as γ is increased from zero,. Certainly for small γ<<1, $N_i^* \approx 1 > 0$ and as such, κ = (1-cT)>0. Because the term (*1-cT*) is common to all species, and assuming that species do not all take negative population values simultaneously as exhaustively checked numerically, the first single species extinction can only occur when for some species-i the term

$\left(1 - \sum_{j=1}^{n} b'_{ij}\right) = 0$, and κ must be positive at least until this point.

1C. Estimating the Probability of Feasibility: Competition

The feasibility are shown to be purely a function of structural disturbance $\boldsymbol{\gamma}$ (*19*,*20*, 48), as claimed in main text. In preparing the figure below we make use of a higher-order approximation for the abundance of the *i*'th species is given by: $N_i^* = \kappa\ (\,1 - X_i\,)$ where the simplest approximation is

$$X_i = B_i' - \sum_{j=1}^{n} b_{ij}'\, B_j' \quad \text{and } B_i' = \sum_{j=1}^{n} b_{ij}/(1-c) \ \text{ and } \kappa > 0 \qquad (7)$$

(See Stone 1988; Roberts 1989; Roberts and Stone 2004 who derive this more accurate approximation):

The distribution of the X_i is asymptotically normal as $n \to \infty$, but even for n as small as *n=5*, the Normal distribution provided an adequate approximation. A calculation shows (for ($\gamma < 1$):

$$< X_i >= 0, \qquad Var(X_i) \approx \gamma^2(1+\gamma^2) \qquad \text{where} \quad \gamma = \frac{\sqrt{n}\,\sigma}{1-c} \ .$$

Thus p=$Pr(N_i^* > 0) = Pr(X_i < 1) = Pr(Z < \frac{1}{\sqrt{Var(X_i)}})$. (8)

Here *Z* is a Normal variate with *Z~Normal*(0,1). Note that the probability is purely a function of the single aggregated parameter γ i.e., $p = p(\gamma)$. Based on first-order independence of species equilibria, a first order estimate of system feasibility *Pr(Feasible)* is thus given by the probability that all *n*-species equilibria are greater than zero, namely: $Pr(Feasible) = p^n$. (9)

(These results are used to construct the continuous lines in Supplementary Figure 1 below (and Fig.1b main text)). Hence feasible systems require small γ, specifically $\gamma \ll 1$. Equivalently feasibility requires $\rho(B') = \gamma \ll 1$.

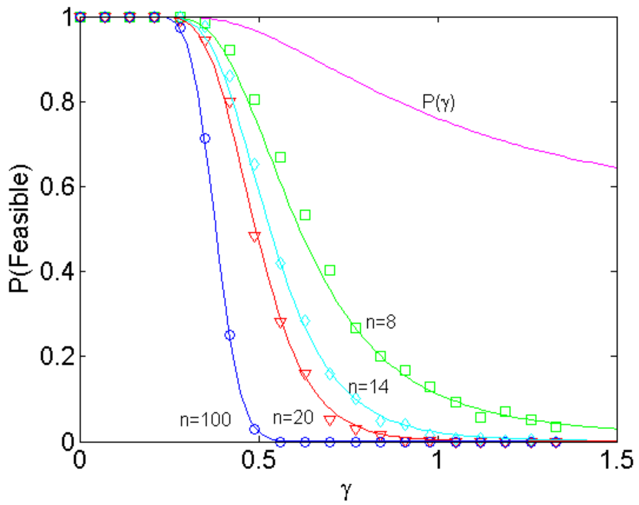

Supplementary Figure 1. *Pr*(feasible) for *n*-species competition communities (*c=0.25*) as a function of structural disturbances γ. Theoretical predictions are given by the continuous curves. Each probability marked by a square, circle, etc is the proportion of feasible systems in 500 runs of Eqn.1 (See also Stone 1988, PhD thesis).

1D. Model stability for systems of competition

The stability matrix is given by $S=DA$ where $D=diag(Ni^*)$ and $A=(a_{ij})$ is the matrix of interactions. Note that $D>0$ for a feasible system with all positive equilibrium populations.

Local Stability: The model equilibrium is said to be locally stable if, when its populations are perturbed from equilibrium by a "small amount", they return to their former steady-state. Following the terminology of RSB, the model is locally stable when all eigenvalues λ_j of S satisfy the condition $\mathrm{Re}(\lambda_j)>0$ for $j-=1,2,...,n$. Local stability of the Lotka-Volterra equations (1) is synonymous with local stability of the stability matrix S (see May 1973).

Global Stability: A feasible system that is globally stable will return to equilibrium no matter what size of perturbation disturbs it. The LV equations are globally stable, if there exists a positive definite diagonal matrix $W>0$, such that the symmetric matrix:

$$G= WA +A^T W>0 \qquad (10)$$

i.e., G is positive definite [see Goh 1977]. Here the matrix A is the interaction matrix. A more restricted test for global stability takes $W=I$, and tests whether $G= A +A^T$ is positive definite. If so, A is globally stable.

It is important to note that when A is globally stable so that condition (10) holds, then so automatically is the stability matrix $S=DA$ if the system is feasible ($D=diag(N^*)>0$). (To see this, choose $W=D^{-1}$ when testing S in *(10).*

D-stability: Assume the matrix A is locally stable. Then A is said to be D-stable if DA is locally stable for all positive diagonal matrices $\mathbf{D}=\mathrm{diag}(d_1\ d_2,..., d_n)>0$. Any matrix A that satisfies (10) is automatically D-stable, and thus DA is locally stable for any $D>0$ (Johnson 1974). Hence if A is D-stable then the stability matrix $S=DA$ is also locally stable at a feasible equilibrium ($D=diag(N^*)>0$).

It is very difficult in general to prove D-stability, and there are still many open questions regarding this property. The RSB paper conjectures that the interaction matrix is always D-stable in the feasible regime for the CM model (SI-2).

1E The Google matrix and discarding the C matrix:

Let $\boldsymbol{A}$ be an nxn matrix subject to a structural perturbation. This is generally formulated by writing the perturbed matrix as $\boldsymbol{A}+\epsilon\boldsymbol{B}$, where ϵ is a small perturbation parameter. A rank-one perturbation requires studying matrices of the form $\boldsymbol{A}+\epsilon uv^T$, where $u\ and\ v$ might be arbitrary n-dimensional vectors. An interesting situation occurs when $u\ \ or\ v^T$ are themselves eigenvectors of $\boldsymbol{A}$, and the "Google matrix can be considered as a special case of the specially low rank perturbed matrix" (Zhou 2011).

The Google matrix A general Google matrix (Ding and Zhou 2007, Horn and Serra-Capizzano 2008) is of the form

$$\boldsymbol{G} = (1-c)\boldsymbol{A} + c\, uv^T$$

where $\boldsymbol{A}$ is an nxn real matrix and u is a right eigenvector of $\boldsymbol{A}$, and the "damping factor" c is in the range $0 < c < 1$. An important property concerns the eigenvalues of $\boldsymbol{G}$. Specifically, if $\boldsymbol{A}$ has eigenvalues $\lambda_1, \lambda_2, .., \lambda_{n-1},\ \lambda_n,$

Assuming the eigenvector u is associated with $\lambda_1,$ then the eigenvalues of $\boldsymbol{G}$ are (Ding and Zhou 2007, Horn and Serra-Capizzano 2008):

$$(1-c)\lambda_1 + v^T u, (1-c)\lambda_2, .., (1-c)\lambda_{n-1},\ (1-c)\lambda_n.$$

This is examined in Stone (1998). (See also Supplementary Note 4). Over the last decade there has been considerable mathematical research into the properties of the Google matrix. More details are given in eg Ding and Zhou (2007), Horn& Serra-Capizzano (2008) Cicone A. Serra-Capizzano (2010) and Supplementary Note 4. In the case of the LV competition system the interaction matrix is:

$$\boldsymbol{A} = (1-c)\boldsymbol{I} + B \quad + C \ = \ (1-c)[\boldsymbol{I} + \boldsymbol{B}'] + c\boldsymbol{U}, = (1-c)[\boldsymbol{I} + \boldsymbol{B}'] + c\ e.e'$$

where the matrix of ones is $\boldsymbol{U} = e.e'$ and. $e' = [1,1,1, \ldots.,1,1]$. But $\boldsymbol{A}$ is *not* a Google matrix since e is not an eigenvector of $\boldsymbol{A}$.

__Claim: The community matrix *S*=*DA* has the key properties of a Google matrix.__

To see this, note that $\boldsymbol{S}$ can be written in the form

$$S = DA = (1-c)D[I + B'] + cDU = \quad (1-c)D[I + B'] + cN^*e',$$

Also note that at equilibrium $AN^* = ADe = e,$ and thus in our case $\boldsymbol{AD}$ is a row stochastic matrix. Also note $SN^* = DAN^* = De = N^*$ Thus the vector $N^*$is a right eigenvector of $\boldsymbol{S}$**.** All we need to show is that the vector N^* is a right eigenvector of $D[I + B']$.

Now: $SN^* = D[(1-c)[\boldsymbol{I} + \boldsymbol{B}'] + c\boldsymbol{U}]N^* = N^*$**.**

So $D(1-c)[\boldsymbol{I} + \boldsymbol{B}']N^* + cD\boldsymbol{U}\, N^* = N^*$

If we let $T = \sum_{i=1}^{M} N_i^*$, then where $D\boldsymbol{U}\, N^*$=TN^*

then $D(1-c)[\boldsymbol{I} + \boldsymbol{B}']N^* \ = (1 - \mathrm{cT})N^*$ (10b)

Since N^* is a right eigenvector of $D[I + B']$, then $\boldsymbol{S}$ is of the same form as a Google matrix.

Eigenvalue properties of Google Matrix

A special feature of the Google matrix is its eigenvalue properties. I now proceed to describe one of the main properties, as it relates to our work. For the competition system, the stability matrix S may be written as:

$$S = DA = D[(1-c)I + B + C] \ . \qquad (11)$$

Consider the reduced matrix: $$S^{\dagger} = DA^{\dagger} = D[(1-c)I + B]. \qquad (12)$$

From eqn.10b we see that $S^{\dagger}$has an eigenvalue $(1 - cT)'$associated with the right eigenvector N^* .

<u>**1F** Suppose S has the n eigenvalues</u> $\boldsymbol{\lambda_1, \lambda_2, .., \lambda_{n-1}, \lambda_n = 1}$ **. where the spectral radius of S is** $\boldsymbol{\rho(S) = \lambda_n = 1}$

Claim (see Stone 1988 PhD thesis copied in SN4):

$S^{\dagger}$ **has (n-1) identical eigenvalues** $\boldsymbol{\lambda_1^{\dagger} = \lambda_1,\ \lambda_2^{\dagger} = \lambda_2, .., \ \lambda_{n-1}^{\dagger} = \lambda_{n-1},}$ **to** $\boldsymbol{S}$ **except for**

$$\lambda_n^{\dagger} = 1\text{-}cT \qquad where\ \ T = \sum_{i=1}^{M} N_i^* \qquad (13)$$

Proof: Suppose *S*=*DA* has eigenvalues $\lambda_1, \lambda_2, .., \lambda_{n-1},\ \lambda_n = \rho = 1,$ where the unity spectral radius ρ derives from the equilibrium condition *AN**=*e*. Thus *S*=*DA* satisfies the equation *SN**=1*N** and has the right eigenvector *N** associated with the real eigenvalue λ=+1 (also the spectral radius of *S*). .

Note that the columns of the "perturbation matrix" *DC* consists of identical columns containing the right eigenvector cN^* of *S*. We use the fact that the left eigenvectors of a matrix are orthogonal to their non-corresponding right eigenvectors (Aitken 1958). In particular, if we let $\underline{v}_i$ represent the left eigenvectors of *S*, then $\underline{v}_i^T . N^*=0$, where $\underline{v}_i^T$ corresponds to any eigenvalue $\lambda \neq \rho = 1$.

Note that: $$(v^T.S)_i = (v^T.S^{\dagger})_i + cv^T.N^* = (v^T.S^{\dagger})_i \qquad (\text{since } \underline{v}_i^T . N^*=0).$$

Thus $$v^T.S = v^T.S^{\dagger} = \lambda v^T \qquad (14)$$

Hence *S* and $S^{\dagger}$ have the eigenvalue λ in common.

To illustrate the point, consider the stability matrix **S=*D*(**$A^{\dagger}$**+*cU*)** where ***U*** has entries all u_{ij}=1 and $A^{\dagger}$ is the modified May matrix $A^{\dagger} = I + B$. (Note that we deliberately do not set $A^{\dagger} = (1-c)I + B$ to remove the confounding effect of competition via the (1-c) term which would interfere with our goal here.) The matrix $A^{\dagger}$ is here perturbed by the rank-one matrix C. Supplementary Figure 2 below plots the real part of the critical eigenvalue of S $(i.e., \min_i Re\{\lambda_i(S)\})$ as a function of c. The eigenvalue does not change with c.

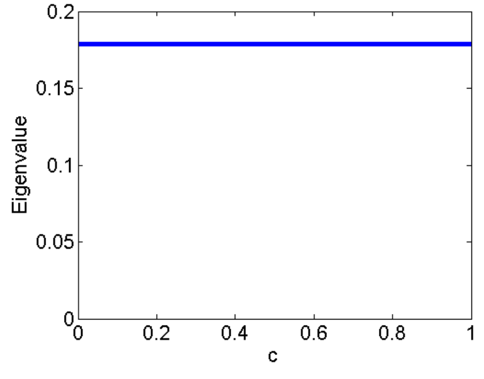

Supplementary Figure 2 . n=10;c=0.4; spread v=0.4

The competition coefficient c in matrix $A = D(I + B + C)$ fails to affect the system's critical eigenvalue.

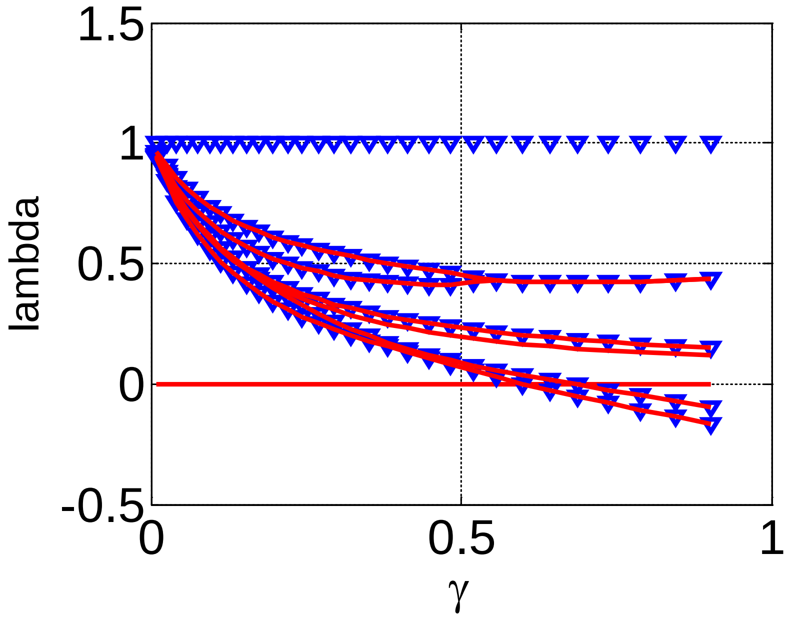

n=10; m=0.4;v=0.4;

Supplementary Figure 3 see below

<u>Identical eigenvalues of S and $S^{\dagger}$ in competition system:</u> As a final illustration, Supplementary Figure 3 above plots the real parts of all six eigenvalues of $S^{\dagger} = D[(1-c)I + B]$ having $Re\{\lambda^{*}(S^{\dagger})\}$ (red lines), and the real parts of eigenvalued of $S = D[(1-c)I + B + C]$ (blue triangles) as a function of c. All eigenvalues are identical except for one. Note that matrix S has spectral radius λ=1 always, while $S^{\dagger}$ has a corresponding Perron eigenvalue λ=1-cT (The red line at lambda=0 only highlights the x-axis.)

<u>Important Note:</u> It is important to emphasise that the two matrices S and $S^{\dagger}$ share (n-1) eigenvalues, whether or not B is a random matrix, and exactly for all matrix sizes *n*. The effect is independent of random matrix theory. We take advantage of this later in our study of empirical mutualistic networks in Supplementary Note 3.

<u>1G. The interaction matrix A is both globally and D-stable for feasible systems when $\gamma < \sqrt{2}/2$=0.71</u> <u>(Stone 1988).</u> *Thus S=DA* is globally stable for feasible systems when $< \sqrt{2}/2$.

To show this, we make use of Wigner's (1967) semi-circle law: If the elements of the Hermitian matrix *A* are independently chosen from some statistical distribution such that for all i,j $a_{ij} = a_{ji}$*, $<a_{ij}>$=0 and Var(a_{ij}) =σ^2, then the eigenvalues which are all real, are distributed as n→ ∞, according to the semi-circle law, with density *d(x),* where

$$d(x)=\begin{cases} \frac{\sqrt{(4n\sigma^2-x^2)}}{2n\pi\rho^2} & for|x| < 2\sqrt{n}\sigma \\ 0 & for\ |x| > 2\sqrt{n}\sigma \end{cases} \qquad (15)$$

For our case, consider $A^{\dagger} = [(1-c)I + B]$. Here the b_{ij} are random perturbations having mean zero and variance Var(b_{ij})=σ^2. Form the matrix

$$G=\frac{1}{2}(A^{\dagger} + A^{\dagger T}),$$

where $g_{ii}=1-c$, and $g_{ij}=\frac{1}{2}(b_{ij} + b_{ji})$, so that Var($g_{ij}$)= $\sigma^2/2$. We use Eq.10 in SI-1D and Eq.15 to determine global stability, and find that all eigenvalues of G are positive, and thus $A^{\dagger}$ is globally lyapunov stable, if $\gamma < \frac{1}{\sqrt{2}}$=0.71 .

The interaction matrix may be written as $A = A^{\dagger} + cU$ where U is the nxn matrix whose elements are all u_{ij}=1. will also be globally stable if $\gamma < \frac{1}{\sqrt{2}}$=0.71. This follows since $A+A^T = A^{\dagger} + A^{\dagger T} + 2cU > 0$ is then also positive definite, given the matrix U is positive semi-definite (with one eigenvalue *n*, and (*n-1*) eigenvalues zero). Hence *A* is globally stable [using *W=I* in Eq.10 SI-1D]

Since the interaction matrix *A* globally stable when $\gamma < \sqrt{2}/2$=0.71, so too is the stability matrix *S=DA* for feasible systems (by (10) which implies that in this regime, globally stable interaction matrices A are D-stable).

This result that *A* is globally stable when $\gamma < \sqrt{2}/2$=0.71, has been confirmed by numerical simulations and can be visualised in the figures provided in section SN1-J.

<u>May-Wigner-Girko theorem for real random matrices:</u>

The stability properties of the matrix of fluctuations $A_M = (1-c)I + B$ have been characterised by May *(1,2).* Here A_M is a real random matrix where the b_{ij} are random perturbations having mean zero and variance Var(b_{ij})=σ^2. The eigenvalues of A_M are distributed uniformly in a disk in the plane centred at (1-c,0i), and of radius $\sqrt{n}\sigma$.

Let λ_1 be the critical eigenvalue of minimum real part. We see that $Re(\lambda_1) \cong$1-c- $\sqrt{n}\sigma$ and λ_1 sits at the left extreme of the disk at coordinates (1-c- $\sqrt{n}\sigma$,0i). Hence May argued that large complex systems (though unstructured by competition) can only be stable when $Re(\lambda_1)$>0, meaning the interaction disturbances are "not too large," namely when: $\boldsymbol{\gamma} < 1$, where $\gamma = \frac{\sqrt{n-1}\,\sigma}{1-c}$

1H) Feasibility is lost before stability of the interaction matrix A.

The approach used here assumes we begin with a feasible stable competition-system, and suppose that a destabilizing parameter such as $\boldsymbol{\gamma}$ is continually increased from zero. We then argue that the systems will lose feasibility before the interaction matrix becomes unstable. To see this we first examine what happens close to where determinant $|A| = 0$,

a) For competition-systems there is population blow up as $|A| \to 0$.

We are interested in exploring the model when $|A| \to 0$. Let the vectors λ and λ' be two different rows of the interaction matrix A, and λ_i correspond to the i'th component in the vector λ. When solving for equilibrium, after scaling we have an equation of the form $Ax=b$. Without loss of generality, two rows of this equation would read:

$$\sum r_i x_i = 1$$

$$\sum r_i' x_i = 0$$

Hence

$$\Sigma(r_i - r_i')x_i = 1$$

A zero determinant $|A| = 0$, is equivalent to having two identical rows in A, say r and r'.

Now $1 = \| (r - r')x \| \leq \| r - r' \| \| x \|$

Let $\varepsilon = \| r - r' \|$. Then as $\varepsilon \to 0$, this implies $\| x \| \geq \frac{1}{\varepsilon} \to \infty$. (16)

Hence as $|A| \to 0$, at least one equilibrium population must blow up, say $N_1^* \to \pm\infty$, which is what happens in practice (Fig.S1-3). Now consider the structure of the competition equations (Eqn.1) at equilibrium. For the first population, for example, at equilibrium:

$$(1-c)N_1^* + \sum_{j=1}^{n} a_{ij} N_j^* = 1 \qquad \text{where } a_{ij} > 0. \qquad (17)$$

b)The equations ensure that if one population $\boldsymbol{N_1^*} \to +\infty$, then this must be counterbalanced by another population, say $\boldsymbol{N_2^*} \to -\infty$. This means the system must become unfeasible when $|A| \to 0$,.

We just saw that population blow up will destroy feasibility. Clearly this will happen if, as γ is increased from zero, if either:

1) **the smallest eigenvalue of A is real** and changes from positive to negative when it passes through $\lambda_n = 0$. . This will ensure blowup because $|A| = \prod \lambda_j$ and the determinant passes through $|A| = 0$ as it changes from positive to negative

2) **the smallest eigenvalue has an imaginary component that is relatively small**. In that case, $|A| \cong 0$ (but not zero) as the real part of a pair of two complex conjugate eigenvalues change from positive to negative. This is the more difficult situation to deal with.

In both these scenarios **feasibility is lost before stability of the interaction matrix A is lost.**

Examples and explanation:

i) **<u>The critical eigenvalue is real and</u>** $|A| = 0$ when the critical eigenvalue of A changes from positive to negative as a parameter is varied.

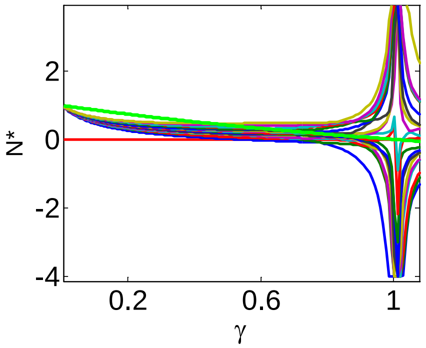

Supplementary Figure 4. k=20 v=0.7

In Supplementary Figure 4 above, the green line plots $Re(\lambda_1)$, the real part of the critical eigenvalue λ_1 of the interaction matrix, as a function of parameter c. Checks show $Im(\lambda_1) = 0$. Strong unmistakable population blow up occurs when $Re(\lambda_1) = \lambda_1 = 0$ or in this case, equivalently $|A| = 0$ at $\gamma \simeq 1$ i.e., when the real eigenvalue changes from positive to negative**.** Feasibility is lost when $\gamma \simeq 0.58$ as the populations begin to explode, before stability is lost at $\gamma = 1.04$.

ii) We examine that subset of cases in which $Im(\lambda_1) \neq 0$ while the real part of this critical eigenvalue changes from positive to negative**.** In Supplementary Figure 5 below, the green line plots $Re(\lambda_1)$ while the cyan line plots $Im(\lambda_1)$ of the critical eigenvalue λ_1 of the interaction matrix A, as a function of parameter γ.

Stability is lost when $Re(\lambda_1)$ of the eigenvalue of A having smallest real part changes from positive to negative. At this point $|A| \cong 0$ (but does not zero because the eigenvalue is complex and not zero), but we still expect populations to attain intermediate to large magnitudes (see figure below), both positive and negative, for γ close to where $|A| \cong 0$. As **a result, populations are dragged negative** , for γ **well before $|A| \cong 0$, and thus feasibility is lost before the stability of the interaction matrix A is lost.**

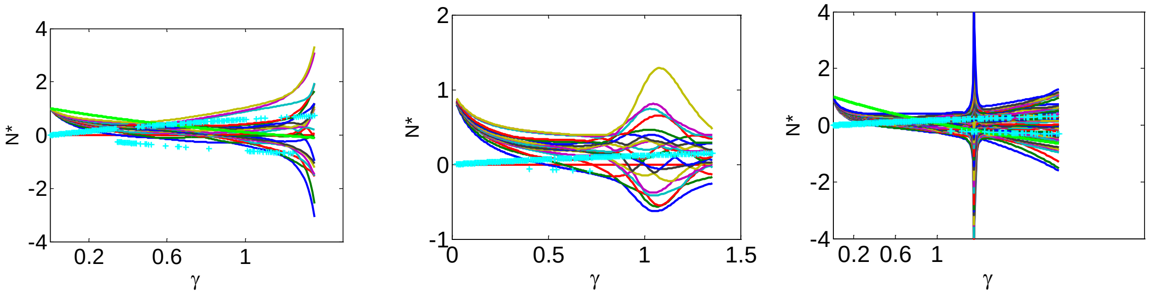

Supplementary Figure 5 a) k=20 v=1 b) k=20 v=1 c) k=50 v=1

As discussed in the last sections, the May-Wigner semi-circle law posits that the interaction matrix A has eigenvalues that are uniformly distributed in a disk in the complex plane. The critical eigenvalue λ_1 is located on the far "left-hand" side of the disk close to the origin. As n →∞, the disk becomes denser with eigenvalues, and when $Re\{\lambda_1\} = 0$ the imaginary component of the critical eigenvalue $Im\{\lambda_1\}$ →0. As such $|A|$→0 thereby magnifying the populations (Panel c in Supplementary Figure 5).

This last argument is valid for large n. However for small n, when the eigenvalue having smallest real part nevertheless has a large imaginary component, this line of argument may have limitations as seen in the middle panel of Supplementary Figure 5. Here, there is only minor population blowup when $Re\{\lambda_1\} \rightarrow 0$.. This is usually enough to explain the epidemic like growth in both positive and negative equilibrium population levels responsible for feasibility loss. In these cases we can also appeal to our feasibility calculations summarised in SN1-D. There we showed that feasibility is only plausible when $\gamma < 1$ and for large systems $\gamma \ll 1$.

SN1-I ***Feasible competition systems are locally stable (i.e. S=DA is locally stable), when May's matrix $A^\dagger$ is locally stable, namely when $\gamma < 1$, and unstable otherwise***. This is true for large systems, although exceptions may occur for smaller sized systems eg n<30, but in practice even exceptions are rarely observed.

i) Section G proves that feasible competition systems are locally and globally stable, when May's matrix $A^\dagger$ satisfies $\gamma < \frac{1}{\sqrt{2}}$=0.71, which covers most of the range of nearly all feasible systems, and most likely all of the range for large systems given the feasibility constraint Eqn.9.

ii) We now explore wider conditions. To begin, recall May's work which considered matrices of the form $A^\dagger = [(1-c)I + B]$. Here the b_{ij} are random perturbations having mean zero and variance $\mathrm{Var}(b_{ij}) = \sigma^2$. Suppose the disturbance γ is gradually increased eg., by increasing σ . May showed the matrix $A^\dagger$ will transition from stability to instability when $\gamma = 1$ (see main text and SI-1B&F). **Here we need to show that $S^\dagger$=D$A^\dagger$ also follows this transition for feasible systems (*D*>0).**

Note that from property in **F**) above, the critical eigenvalue of S and $S^\dagger$ are exactly the same. Therefore it is sufficient to study stability by examining the critical eigenvalue of $S^\dagger = DA^\dagger = D[(1-c)I + B]$.

The eigenvalues of $A^\dagger$ are distributed uniformly in a disk in the complex plane (May-Wigner theorem) centred at ((1-c),0i) of radius $\sqrt{n}\sigma$. The eigenvalue having minimum real part, $\lambda_1 \cong 1 - \mathrm{c} - \sqrt{n}\sigma$, sits at the left extreme of the disk close to the coordinates (1-c- $\sqrt{n}\sigma$,0i).

As before, two cases need to be considered for feasible systems (*D*>0) depending on whether the critical vaue of the eigenvale is real or complex:

Case a) The critical eigenvalue λ_1 of $A^\dagger$ is real. As σ is increased, λ_1 changes from positive to negative crossing through $\lambda_1 = 0$ at the stability threshold $\sigma = \sigma_c$. At this point $|A^\dagger| = \prod_j \lambda_j\,(A^\dagger) = 0$. Thus, at the stability transition

$$|S^\dagger| = |D||A^\dagger| = |D| \prod_j \lambda_j\,(A^\dagger) = \prod_j \lambda_j\,(S^\dagger) = 0,$$

and thus the critical eigenvalue of $S^\dagger$, $\lambda_1(S^\dagger)$=0. Hence $S^\dagger$ loses stability exactly when $A^\dagger$ loses stability at $\sigma = \sigma_c$.

Case b) The critical eigenvalue $\lambda_1(A^\dagger)$ having smallest real part, also has a component that is imaginary. This makes many difficulties for any analysis. As σ increases, $Re\{\lambda_1(A^\dagger)\}$ changes from positive to negative crossing through $Re\{\lambda_1(A^\dagger)\} = 0$ at the stability threshold $\sigma = \sigma_c$. However, unlike previously, at this threshold point $|A^\dagger| = \prod_j \lambda_j\,(A^\dagger) \neq 0$.

However, according to the May-Wigner theory, the critical eigenvalue $\lambda_1(A^\dagger)$ will be found on the far "left-hand" side of the disk containing the eigenvalues in the complex plane. As n $\rightarrow\infty$, the disk becomes denser with eigenvalues, and when $Re\{\lambda_1(A^\dagger)\} = 0$, the imaginary component of the critical eigenvalue $Im\{\lambda_1(A^\dagger)\} \rightarrow 0$. As such $|A^\dagger| \rightarrow 0$, and since $|S^\dagger| = |D||A^\dagger|$ we see that also $|S^\dagger| \rightarrow 0$. For this reason, in large systems $A^\dagger$ and $S^\dagger$ lose stability simultaneously or very close to simultaneously at the stability threshold $\sigma = \sigma_c$.

CORROLLARY: The particular eigenvalue of $S^{\dagger} = DA^{\dagger}$, namely λ=1-cT , is positive for $\gamma < 1$ (as required in Section SN1-B) for large feasible systems.

1J) Feasibility and stability for communities with *n=8,14 &20 species (upper, lower graphs* Supplementary Figure 6*)* as a function of γ, where c=0.3, based on an analysis of 1,000 random matrices for each point in the graph *(cf Fig.1b main text).* The graphs display the percentage of feasible systems %F (red); a) the percentage of interaction matrices A that were found locally stable %L(A) (blue); the percentage of feasible systems with locally stable interaction matrices A (%F&L(A)) and stability matrices S (%F&L(S)). b) the percentage of globally lyapunov stable interaction matrices %G(A) (blue); percentage of feasible systems with globally stable interaction matrices %F&G(A).(green). Here %G(A) is the percentage of matrices A such that $A+A^T>0$ (positive definite) and thus globally stable. These matrices satisfy condition in Eq.10 (SN1-D) where *W=I.*

n=8 (a) (b)

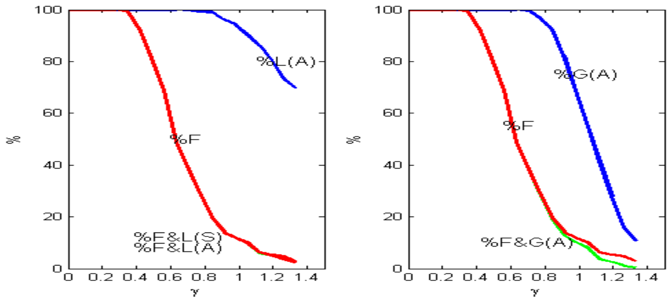

***n*=14 (a) (b)**

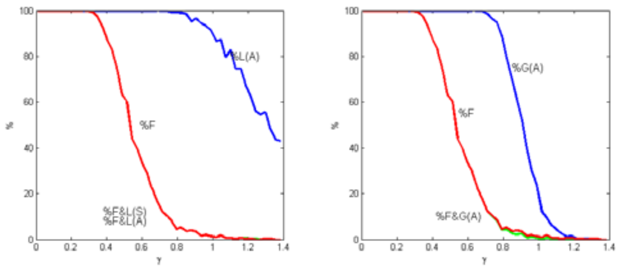

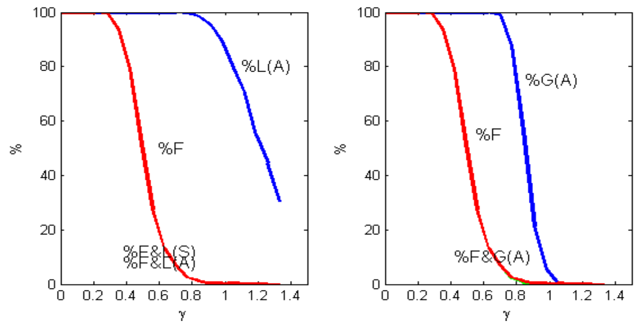

***n*=20 (a) (b)**

Supplementary Figure 6 . Feasibility and stability for communities with *n=8&20* species as a function of γ, where c=0.3, based on an analysis of 1,000 random matrices for each point in the graph.

1K RANK-1 Perturbations

It is shown that the critical (smallest) eigenvalue of the stability matrix S is completely unaffected by rank-one perturbations (addition of the C* matrix) in the LV competition equations (SN1- Eqn1):

Critical Eigenvalue of the 20x20 Stability Matrix S=*D(A+cU)* for nine different values of c over interval (0,1). The nine eigenvalues, listed below are exactly constant for all c as predicted by the theory. Here D=diag(N*)>0

0.024090888644307	0.024090888644307	0.024090888644307	0.024090888644307
0.024090888644307	0.024090888644307	0.024090888644307	0.024090888644307
0.024090888644307			

It is shown that the critical eigenvalue of the stability matrix S is largely unaffected by rank-one perturbations (addition of the C* matrix), even when the carrying capacities k(i) are not all equal to unity in the LV equations (SN1- Eqn1):

$$\frac{dN_i}{dt} = r_i N_i (k_i - \sum_j a_{ij} \, N_j)$$

Critical eigenvalues of the 10x10 matrix S=D(A+cU) with heterogeneous carrying capacities k_i of i'th species is k_i =1+0.25 rand(i) (where rand is uniform random variable in interval (0,1)).

Now the critical eigenvalue of the matrix S, listed below is almost constant for 30 different values of $0<c<1$, but deviates slightly (5'th decimal place).

0.046415239056272	0.046417337756125	0.046419372583605	0.046421346408414	0.046423261931022
0.046425121694953	0.046426928098010	0.046428683402561	0.046430389744959	0.046432049144186
0.046433663509802	0.046435234649253	0.046436764274602	0.046438254008735	0.046439705391088
0.046441119882940	0.046442498872310	0.046443843678485	0.046445155556223	0.046446435699645
0.046447685245857	0.046448905278305	0.046450096829909	0.046451260885964	0.046452398386861
0.046453510230610	0.046454597275199	0.046455660340802	0.046456700211834	0.046457717638884

Carrying capacities k(i)=: 1.052269167805285 1.110192847811202 1.077768507705748 1.224673153998787 1.216582001804424 1.018972535601082 1.103097521452674 1.227020547290352 1.022724169719322 1.232148553825014

Supplementary Note 2: Competition-Mutualist (CM) Model

2A. Preliminaries

As in RSB (Rohr et al. (2014)), let P_i and A_i denote the abundances of plant and animal species-i respectively. The competition- mutualist CM-model, Eqns.7 of the main text, are:

$$\frac{dP_i}{dt} = P_i(1 - \sum_j c_{ij}^{(P)} P_j + \sum_j m_{ij}^{(P)} A_j) \qquad \frac{dA_i}{dt} = A_i(1 - \sum_j c_{ij}^{(A)} A_j + \sum_j m_{ij}^{(A)} P_j) \quad . \tag{2.1}$$

Similar to the uniform competition model, all plants species compete with each other with the same negative interaction strength c, and likewise for animal species so that $c_{ij}^{(P)} = c_{ij}^{(A)} = c < 1\ (i \neq j)$. Intraspecific competition is scaled to unity so that $c_{ii}=1$.

As in RSB, the mutualistic benefit:

$$m_{ij} = (m_{ij}^0 y_{ij})/k_i^\delta \tag{2.2}$$

where $y_{ij} = 1$ if species-i and -j interact and zero otherwise. k_i is the number of mutualistic interactions of species-i, m_{ij}^0 represents the level of mutualistic strength. The parameter δ corresponds to a mutualistic trade-off that modulates the extent to which a species that interacts with few other species does it strongly, whereas a species that interacts with many partners does it weakly. Our methodology works for both $\delta = 0$ (no benefit) and with a strong mutualistic benefit $\delta = 1$ (see eg., SN3 Fig.1).

In general, for the stochastic ensemble model and for nonzero interactions, we let $m_{ij}^0 = m + b_{ij}$. As before, the b_{ij} are selected randomly (uniformly) from the interval $[-mv, +mv]$ where $0 \leq v \leq 1$, so that

$$< b_{ij} > = 0 \text{ and } Var(b_{ij}) = \frac{m^2 v^2}{3} = \sigma^2 .$$

This differs from RSB who set mutualistic interactions, if they occur, to the same constant m. We also allow for incorporation of community connectance whereby only a proportion q of the m_{ij} are non-zero.

The scheme above requires an interaction matrix that has two diagonal blocks (C^* shown below) to describe the background skeleton network of competitive interactions within plants and within animals separately. Additionally there are two off-diagonal cooperative blocks which define the network of mutualistic interactions from animals to plants, and vice-versa.

The interaction matrix $\boldsymbol{A}$ may be split into its competitive and mutualistic components:

$$A = \begin{bmatrix} 1 & c & 0 & 0 \\ c & 1 & -m & 0 \\ 0 & -m & 1 & c \\ 0 & 0 & c & 1 \end{bmatrix} = (1-c)I - M^* + C^*$$

Mutualistic M^* Competition C^*

A typical competition matrix C^* for n=10 species ($n_1 = n_2 = 5$) is the following:

$$\begin{matrix} 1 & 1 & 1 & 1 & 1 & 0 & 0 & 0 & 0 & 0 \\ 1 & 1 & 1 & 1 & 1 & 0 & 0 & 0 & 0 & 0 \\ 1 & 1 & 1 & 1 & 1 & 0 & 0 & 0 & 0 & 0 \\ 1 & 1 & 1 & 1 & 1 & 0 & 0 & 0 & 0 & 0 \\ 1 & 1 & 1 & 1 & 1 & 0 & 0 & 0 & 0 & 0 \\ 0 & 0 & 0 & 0 & 0 & 1 & 1 & 1 & 1 & 1 \\ 0 & 0 & 0 & 0 & 0 & 1 & 1 & 1 & 1 & 1 \\ 0 & 0 & 0 & 0 & 0 & 1 & 1 & 1 & 1 & 1 \\ 0 & 0 & 0 & 0 & 0 & 1 & 1 & 1 & 1 & 1 \\ 0 & 0 & 0 & 0 & 0 & 1 & 1 & 1 & 1 & 1 \end{matrix} \;.\; c \;=\; C^*$$

The matrix C^* is rank-2 and can be written as a sum of two rank-one matrices, namely:

$$C^* = c\, e_1.e_1^T + c\, e_2.e_2^T ,$$

where e_1=[1 1 1 1 1 0 0 0 0 0] and e_2 =[0 0 0 0 0 1 1 1 1 1]. (C^* is positive semi-definite having two positive eigenvalues and the remainder zeroes.)

The Mutualism Matrix *M (not necessarily symmetric)*

Suppose $\mathcal{M}$ is an px(n-p) matrix and $\mathcal{M}^T$ is thus an (n-p)xp matrix. Then the *n*x*n* matrix

$M' = \begin{bmatrix} 0 & -\mathcal{M} \\ -\mathcal{M}^T & 0 \end{bmatrix}$ is symmetric and bipartite. Note the matrix $(M')^2$ has the same real eigenvalues as the product $\mathcal{M}\mathcal{M}^T$, so that the eigenvalues of $M'$are the square roots of these numbers. Thus the matrix M' has real eigenvalues in pairs $\pm s_i$.

Consider the case when $\mathcal{M}$ is a *p*xn random matrix where *(p<n)*, with entries m_{ij} having mean zero and variance Var(m_{ij})=σ^2. $\mathcal{M}\mathcal{M}^T$has maximum eigenvalue which almost surely is: $(1+\sqrt{y})^2 n\sigma^2$ where *y=p/n*. (Geman 1980). Therefore the maximum eigenvalue of M' is $(1+\sqrt{y})\sqrt{n}\sigma$.

To model the mutualistic benefits described in RSB, we need $D_{1+2} = diag(D_1, D_2)$ where

$D_{1,2}(i)$ =diag$\{1/k_i^\delta\} > 0$. In our context the non-symmetric mutualism matrix M is

$$M = D_{1+2}M' = \begin{bmatrix} 0 & -D_1\mathcal{M} \\ -D_2\mathcal{M}^T & 0 \end{bmatrix} = \begin{bmatrix} 0 & M_1 \\ M_2 & 0 \end{bmatrix}$$

In this study the following cases have been examined:

- "all-to-all" interactions, in which all animals have mutualistic interactions with all plants, whereby $m_{ij} > 0$ for all *i,j*.
- As above, except allowing the mutualism network to have connectance q (which is the probability that any interaction is nonzero).
- empirically observed mutualist pollination networks of arbitrary topology (see main text).

Claim: The bipartite matrix M has eigenvalues that are purely real, by Taussky's theorem:.

Proof: If $A = S_1 S_2$ is the product of two real symmetric matrices, one of them S_1 say, positive definite, then A has real characteristic roots and these roots have the same signs as the roots of S_2. (Taussky 1963). In our case, let $S_1 = D_{1+2}$ be a diagonal matrix and.$S_2 = M'$. Then the eigenvalues of $M = D_{1+2}M'$ must be real.

Similarly, the eigenvalues of $\boldsymbol{A}^{\dagger} = \boldsymbol{I}(\boldsymbol{1}-\boldsymbol{c}) - \boldsymbol{M}$ must be real, and may be ordered

$$\boldsymbol{\lambda_1}(A^{\dagger}) \le \boldsymbol{\lambda_2}(A^{\dagger}) \le \cdots \le \boldsymbol{\lambda_{n-1}}(A^{\dagger}) \le \boldsymbol{\lambda_n}(A^{\dagger}).$$

Setting $\rho(M)$ as the spectral radius or eigenvalue of largest magnitude of M, the following relationship holds

$$\boldsymbol{\lambda_1}(A^{\dagger}) = 1 - c - \rho(M).$$

<u>**Random Matrix Examples:**</u> Consider matrices $\mathcal{M}$ of size kxk=100x100

The m_{ij} are selected randomly (uniformly) from the interval $[-v, +v]$ where $0 \le v \le 1$, so that

$< m_{ij} > = 0$ and $Var(m_{ij}) = \frac{v^2}{3} = \sigma^2$. The May Wigner theorem predicts maximum and minimum eigenvalues of $\mathcal{M}$ to have real parts of magnitude $\lambda_{1\pm} = \pm\gamma$ $(=\pm\sqrt{k}\sigma)$.

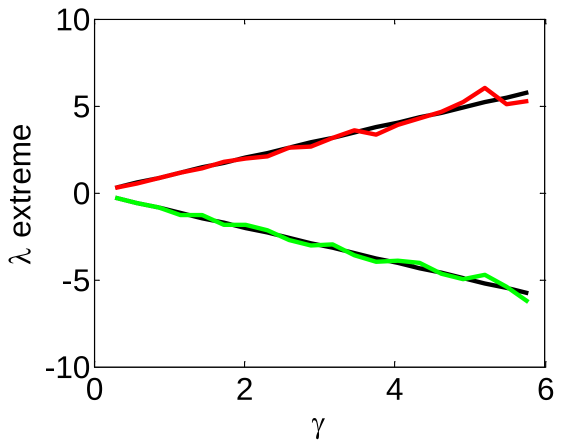

Supplementary Figure 7 $\lambda_{1\pm} = \pm\gamma$ where $\gamma = \sqrt{k}\sigma$ k=100.

Red & green lines from computational analysis of random matrices. Black line is the prediction $\lambda_{1\pm} = \pm\gamma$

(If $\mathcal{M}$ were symmetric then $\lambda_{1\pm/-} = \pm\sqrt{2}\,\gamma$)

Now form the symmetric matrix $M' = \begin{bmatrix} 0 & -\mathcal{M} \\ -\mathcal{M}^T & 0 \end{bmatrix}$ of size nxn=200x200=$2kx2k$. Again the nonzero m_{ij} are selected randomly (uniformly) from the interval $[-v, +v]$ where $0 \le v \le 1$. The eigenvalues are all real.

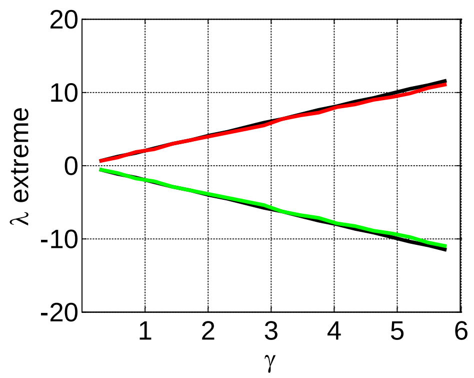

Supplementary Figure 8 $\lambda_{1\pm-} = \pm 2\gamma = 2\sqrt{k}\sigma = \sqrt{2n}\sigma$ $\gamma = \sqrt{k}\sigma$ k=100, where reference $\mathcal{M}$ has size k=100x100) and n=2k=200 is size of matrix M'. Red & green lines from computational determination of $\lambda_{1\pm-}$ from random matrices, Black line is prediction $\lambda_{1\pm/} = \pm 2\gamma$

Modelling CONNECTANCE-q in off-diag blocks of $M' = \begin{bmatrix} 0 & -\mathcal{M} \\ -\mathcal{M}^T & 0 \end{bmatrix}$

Let M' be a nxn bipartite matrix with kxk diagonal blocks zero where k=n/2.
The nxn symmetric off-diagonal blocks have connectance=q. i.e.: q=probability an element is one; (1-q)=probability an element is zero. Each element of $\mathcal{M}$ has mean q/2 and variance q(1-q).
Then as a function of q, the subdominant eigenvalue sits on the line : $\lambda_2 = 2\sqrt{k}\sigma = 2\sqrt{k * q(1-q)}$
As a function of q, the largest eigenvalue sits at λ_1=nq

In Supplementary Figure 9 below k=100

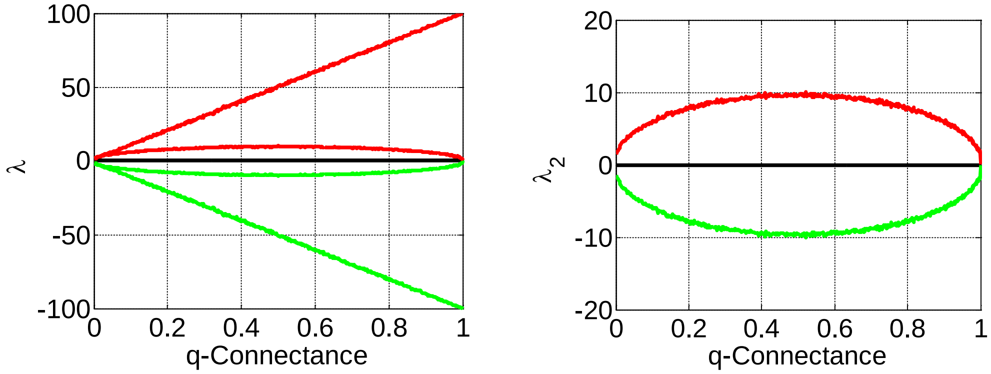

Supplementary Figure 9 n=100

As a function of q, the subdominant eigenvalue sits on the line : $\lambda_2 = 2\sqrt{k}\sigma = \sqrt{400 * q(1-q)}$
As a function of q, the largest eigenvalue sits at λ_1=kq= 100*q

<u>2B. Low Rank Perturbations of the CM-model: In practice</u>

For the CM-model, we are interested in the properties of the stability matrix S and it's relative $S^{\dagger}$:

S=$DA = D[I(1-c) - M + C^{*}$ $\qquad S^{\dagger} = DA^{\dagger} = D(I(1-c) - M)$

In general, since the structurally disturbed CM-model can be considered as a perturbation from the uniform model, stability of the ensemble model requires that the uniform model be stable. We deal with requirements for stability of uniform model in section 2D.

The approach used here assumes we begin with a feasible stable CM-system, and suppose that a destabilizing parameter such as $\rho(\boldsymbol{M})$ or possibly c is continually increased from zero. Then:

Claim: i) For feasible systems, the matrix $\boldsymbol{S} = \boldsymbol{DA}$ loses local stability when the second smallest eigenvalue of $\boldsymbol{S}^{\dagger} = \boldsymbol{DA}^{\dagger}$ zeroes, i.e $\boldsymbol{\lambda_2(S^{\dagger}) = 0}$.

(The smallest eigenvalues $\lambda_1(S^{\dagger})$ is a "ghost eigenvalue" that does not impact the stability of $\boldsymbol{S} = \boldsymbol{DA}$.)

ii) Thus for the feasible CM-model, the matrix $\boldsymbol{S} = \boldsymbol{DA}$ is locally stable when $\boldsymbol{\lambda_2(A^{\dagger})} > 0$ and unstable when $\boldsymbol{\lambda_2(A^{\dagger})} < 0$.

Recall $A^{\dagger} = I(1-c) - M$. Then equivalently, structurally perturbed feasible CM-systems are locally stable if,

$$\boldsymbol{\Lambda_2(M)} < 1 - c \ , \tag{2.5}$$

and unstable otherwise, where $\boldsymbol{\Lambda_2(M)}$ is the 'second largest' or subdominant eigenvalue of $\boldsymbol{M}$. (Here, for convenience, we use slightly different notation to the main text.)

iib) As a corollary, this implies the weaker condition: local stability of S is ensured if $\boldsymbol{S}^{\dagger}$ is stable i.e., $\boldsymbol{\lambda_1(S^{\dagger})} > 0$ or for feasible systems if $\boldsymbol{A}^{\dagger}$ is locally stable i.e., $\boldsymbol{\lambda_1(A^{\dagger})} > 0$.

Verifying these claims requires an understanding of low-rank perturbations. The underlying idea is best presented visually:

S=DA $= D[I(1-c) - M + C^*$ $S^{\dagger} = DA^{\dagger} = D(I(1-c) -$ $M)$

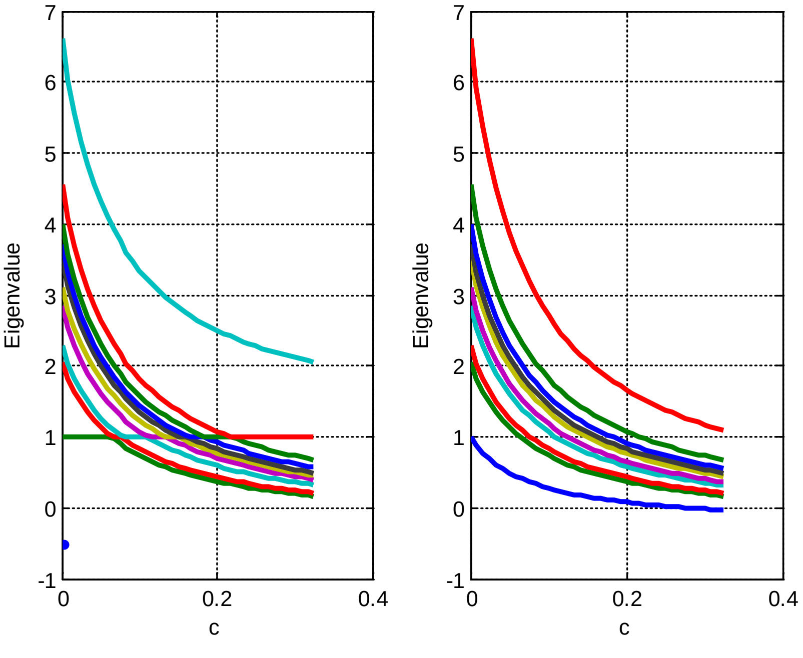

Supplementary Figure 10

The figure above plots the 10 eigenvalues as a function of c for the two stability matrices S (left) and $S^{\dagger}$ (right) for the n=10-species CM-model m=0.14, v=q=1 (all-to-all). The $S^{\dagger}$ matrix has two "outermost" "Perron eigenvalues" in red and dark blue (associated with its positive Perron eigenvector) that are an outcome of its bipartite form. The S matrix has the Perron eigenvalue $\lambda = 1$ which appear as a horizontal line in LH panel connected with the equilibrium condition AN*=1 or SN*=$1N$*.

Supplementary Figure 10 shows that 8 eigenvalues of the S matrix closely approximate the "inner" eigenvalues of $S^{\dagger}$. The other two eigenvalues should be associated with the "outer" Perron roots of $S^{\dagger}$. The S matrix must have an eigenvalue of λ=+1 for all c, as seen in LH panel. This corresponds with the lower-most Perron eigenvalue of $S^{\dagger}$ in the RH panel.

Importantly, the second smallest eigenvalue of $S^{\dagger}$, notated here as $\boldsymbol{\lambda_2}(\boldsymbol{S}^{\dagger})$ approximates well the smallest eigenvalue of *S* for almost the entire range of c.

Hence, Claim i) For feasible systems, the matrix $\boldsymbol{S} = \boldsymbol{DA}$ loses stability, when the second smallest eigenvalue of $\boldsymbol{S}^{\dagger} = \boldsymbol{DA}^{\dagger}$ zeroes, i.e $\boldsymbol{\lambda_2}(\boldsymbol{S}^{\dagger}) = \boldsymbol{0}$. These features are theoretically justified in G in more depth below, and have been confirmed numerically (see below).

Claim ii) for the feasible CM-model, the matrix *S*=*DA*, is locally stable when $\boldsymbol{\lambda_2}(\boldsymbol{A}^{\dagger}) > 0$ and unstable when $\boldsymbol{\lambda_2}(\boldsymbol{A}^{\dagger}) < 0$

To see this, recall that all the eigenvalues of *M* and thus of $A^{\dagger} = I(1-c) - \boldsymbol{M}$ are real numbers.

Importantly, whenever $A^{\dagger}$ has a zero eigenvalue, then so too must $S^{\dagger} = DA^{\dagger}$. This can be deduced from the determinant relation; $|S^{\dagger}| = |D||A^{\dagger}| = |D| \prod_j \lambda_j (A^{\dagger}) = \prod_j \lambda_j (S^{\dagger}) = 0$,

Now, when $\rho(\boldsymbol{M})$ is allowed to increase from zero and passes through $\rho(\boldsymbol{M}) = 1 - c$, the smallest eigenvalue of $A^{\dagger}$, or $\boldsymbol{\lambda_1}(A^{\dagger})$ crosses zero. At this point $|A^{\dagger}| = \prod_j \lambda_j (A^{\dagger}) = 0$. Thus the smallest eigenvalue of $S^{\dagger}$, $\boldsymbol{\lambda_1}(\boldsymbol{S}^{\dagger})$=0, and must pass through zero.

As $\rho(\boldsymbol{M})$ is allowed to increase further, eventually $\boldsymbol{\lambda_2}(A^{\dagger})$ changes from positive to negative crossing $\boldsymbol{\lambda_2}(A^{\dagger}) = 0$. At this point $|A^{\dagger}| = \prod_j \lambda_j (A^{\dagger}) = 0$. And thus the next smallest eigenvalue of $S^{\dagger}$, $\boldsymbol{\lambda_2}(\boldsymbol{S}^{\dagger})$=0, must pass through zero.

Now by *i)* above, the 2nd smallest eigenvalue of $S^{\dagger}$, that is $\boldsymbol{\lambda_2}(\boldsymbol{S}^{\dagger})$, very closely approximates the smallest eigenvalue of *S*. Thus the stability matrix *S*=*DA* also loses stability almost exactly when $\boldsymbol{\lambda_2}(A^{\dagger}) = 0$.

Important Note: It is important to emphasise that the two matrices S and $S^{\dagger}$ share (n-2) eigenvalues, at least to a good approximation, whether or not *B* is a random matrix, and for all matrix sizes *n*. The effect is independent of random matrix theory, which best holds for large *n*. We take advantage of this in our study of empirical mutualistic networks in Fig.3(main text)/SN3.

2C.Theoretical justification of low-rank eigenvalue perturbations

SN-1 examined the effect of the low-rank perturbation matrix C for competition systems exactly. Similar techniques may be used for the CM-model. Beginning with $M = \begin{bmatrix} 0 & M_1 \\ M_2 & 0 \end{bmatrix} = \begin{bmatrix} 0 & -D_1B \\ -D_2B^T & 0 \end{bmatrix}$

As noted M is a bipartite matrix, not necessarily symmetric, comprised of two blocks M_1, a j x $(k\text{-}j)$ matrix, and M_2, a $(k\text{-}j)$xj matrix, as in Eq.4 above. Note that for $x\epsilon\mathbb{R}^j$ *and* $y\epsilon\mathbb{R}^{k-j}$ then

$$M\begin{bmatrix} x \\ y \end{bmatrix} = \lambda\begin{bmatrix} x \\ y \end{bmatrix} \Rightarrow M\begin{bmatrix} x \\ -y \end{bmatrix} = -\lambda\begin{bmatrix} x \\ -y \end{bmatrix}.$$

Hence the eigenvectors of M form pairs $x\oplus y$ *and* $x\oplus -y$ to each eigenvalue λ, and the eigenvalues of M are the real numbers $\pm\lambda_i$ (see SN2-A; Berman and Grone 1988).

As the left-eigenvectors of M, $\begin{bmatrix} u \\ \pm v \end{bmatrix}$, are orthogonal to the right-eigenvectors $\begin{bmatrix} x \\ \pm y \end{bmatrix}$, then we must have

$$u^T.x=0 \quad \text{and} \quad v^T.y=0 \qquad \text{(SN2-4)}$$

for eigenvectors [x,y] and [u,v] and associated with different eigenvalues. λ_p *and* λ_q.

Rank-two perturbations: Consider our rank-2 matrix

$$C^* = c\begin{bmatrix} x \\ 0 \end{bmatrix}.[1,1,1,1,0,0,0,0] + c\begin{bmatrix} 0 \\ y \end{bmatrix}.[0,0,0,0,1,1,1,1],$$

Here, choose $\begin{bmatrix} x \\ y \end{bmatrix}$ to be a (positive) right eigenvector of the bipartite matrix M. We examine the effect of the low-rank perturbation C^* on the eigenvalues of M+C^*. (Recall $A = (1-c)I - M + C^*$)

Given orthogonality of left-right eigenvectors of M, we find:

$$[u,v](M + C^*) = [u,v].M + c[u,v].\begin{bmatrix} x \\ 0 \end{bmatrix}.[1,1,1,1,0,0,0,0] + c[u,v]\begin{bmatrix} 0 \\ y \end{bmatrix}.[0,0,0,0,1,1,1,1] = [u,v].M + 0 + 0 = \lambda_p[u,v]$$

In this case all non-Perron eigenvalues of $M + C^*$ (and interaction matrix A) are unchanged by the matrix C^*, including the critical eigenvalue. This is true whether or not M is a symmetric matrix. Here we have taken advantage of the relation $u^T.x=0$ (see Eqn. SN2-4)

Stability of S.

Claim: The eigenvalues of S are excellent approximations of the eigenvalues of $S^\dagger$, (excluding the two outer "Perron eigenvalues") .

The stability matrix is:

$$S = D((1-c)I - M + C^*) = D((1-c)I - M) + c\begin{bmatrix} N_A^* \\ 0 \end{bmatrix}.[1,1,1,1,0,0,0,0] + c\begin{bmatrix} 0 \\ N_P^* \end{bmatrix}.[0,0,0,0,1,1,1,1]$$

The argument relies on the property that the right eigenvector of S is given by the population equilibria $N^* = \begin{bmatrix} N_A^* \\ N_P^* \end{bmatrix} > 0$ which is positive if feasible. i.e., $SN^* = N^* > 0$.

Now if [u,v] is a left-eigenvector of S, then we see that

$$\lambda[u,v] = [u,v]S = [u,v](D((1-c)I - M + C^*) \cong [u,v]\ D((1-c)I - M) + 0 = [u,v]\ \boldsymbol{S}^\dagger$$

since $u^T.N_A^* \cong 0$ and $v^T.N_B^* \cong 0$. For the latter, now there is no longer strict equality as previously in the related Eqn. SN2-4, because the matrix $D((1-c)I - M + C^*)$ is no longer bipartite.

In practice the eigenvalues of S are excellent approximations of the eigenvalues of $S^{\dagger} = D\left((1-c)I - M\right)$, and largely unaffected by the matrix C^{*}. In practice the approximations are excellent because

i) C^{*} being constructed from the right eigenvector of $S^{\dagger}$ (which has same eigenvectors as S);

ii) the bipartite-like structure of S, which enforces $[u, v] . [N_A^* \; N_P^*]^T = 0$ also leaves $u^T . N_A^* \cong 0$ (see Eqn.SN2-4), confirmed numerically;

Via numerical simulations, the approximation has been found to be excellent in a range of examples for various networks of different degree distributions, as in examples given below and throughout the SN and main text.

__Examples__ Below: n=14 species communities ($n_1 = n_2 = 7$), with mutualistic interactions distributed uniformly in the interval [0,2m] for m=0.05, and m=0.1 assuming a probability of nonzero interaction or connectance is q=1.
LH Panel:Blue star = 2nd smallest eigenvalue of $\boldsymbol{S}^{\dagger} = \boldsymbol{D}\left((\mathbf{1}-\boldsymbol{c})\boldsymbol{I} - \boldsymbol{M}\right)$. Red = smallest eigenvalue of S
RH Panel:Blue star = 2nd smallest eigenvalue of $\boldsymbol{A}^{\dagger} = (\mathbf{1}-\boldsymbol{c})\boldsymbol{I} - \boldsymbol{M}$. Red = smallest eigenvalue of A

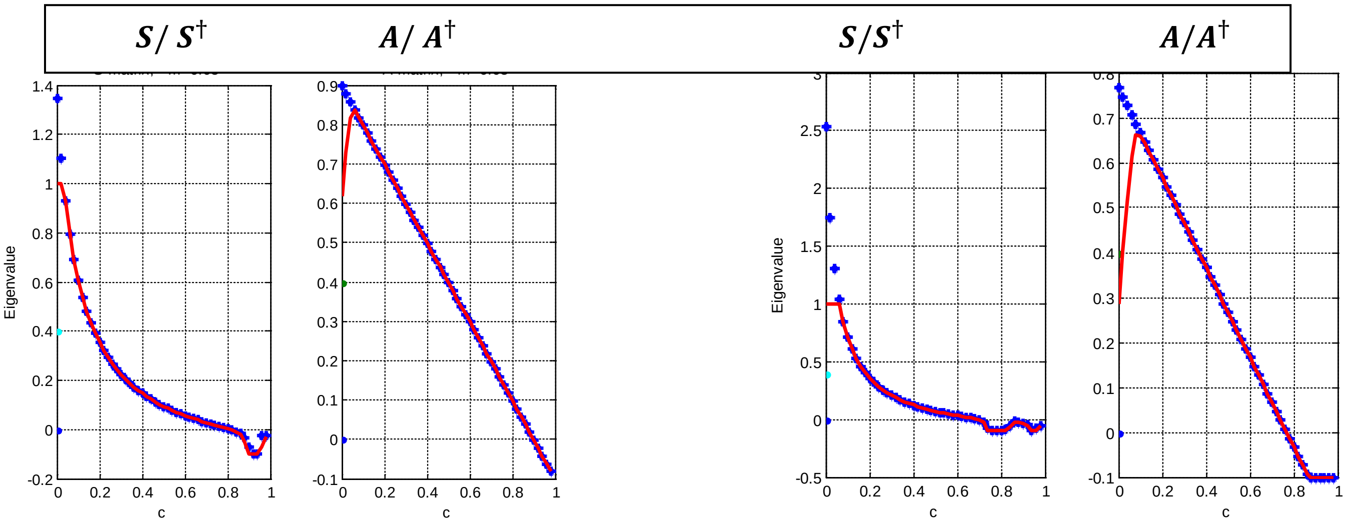

Supplementary Figure 11 **m=0.05** **m=0.1**

Note that the 2nd smallest eigenvalue of $\boldsymbol{S}^{\dagger} = \boldsymbol{D}\left((\mathbf{1}-\boldsymbol{c})\boldsymbol{I} - \boldsymbol{M}\right)$ (blue) and the smallest eigenvalue of S (red) in left-hand panels of Supplementary Figure 11, appear identical for c>m. Also, note the ***precision of the correspondence*** between eigenvalues of S and $\boldsymbol{S}^{\dagger}$, even though these sub-communities are small (n_1xn_2=7x7).

The 2nd smallest eigenvalue of $\boldsymbol{A}^{\dagger} = (\mathbf{1}-\boldsymbol{c})\boldsymbol{I} - \boldsymbol{M}$ (blue) and smallest eigenvalue of $\boldsymbol{A}$ (red) in RH panels also appear identical for most of the domain plotted, for c>m. Differences occurs in the regime when m > c, in accord with the so called LH-border and properties of the BBP phase transition (SN2-D).

__Numerical example__

RANK-2 Perturbations. The animal-plant model of RSB (eqns 7 in main text) are based on an interaction matrix A= (1-c)***I+C+M)*** which has been perturbed with a rank-2 perturbation C*. **n=20; c=0.05; m=0.1;**

Arbitrary eigenvalue of the 20x20 Stability Matrix S=*D(I+C* +M)* for nine different values of c over interval (0,1). The nine eigenvalues, listed below (graphed above in blue, each for a different c value in (0,1)), are almost constant for all c but vary slightly, half a percent, over the full range of c. Here D=diag(N*).

1.7733 1.7766 1.7783 1.7792 1.7798 1.7803 1.7806 1.7808 1.7810

This differs from the true stability matrix [S=*D(I(1-c)+C* +M)] to give the expectation/prediction that the eigenvalue would not change despite changes in the perturbation strength c in the matrix C.*

2D. Finding Left and Right Hand Stability Borders for random CM-model

The BBP (Baik-Ben Arous-Péché (2005)) eigenvalue phase transition: The CM interaction matrix A has unusual stability/instability transitions that arise due to the complex interplay between the low-rank perturbation matrices $C^* - M^*$, and the (off-diagonal block) random matrix B. The BBP theory as expounded and extended in Beynach-Georges and Nadakuditi (2011), posits conditions for when the extreme eigenvalues "separate from the bulk" eigenvalues of a random matrix. This is the same effect described in the previous sections concerning the changes of the Perron eigenvalue, when a low rank perturbation is introduced.

Case 1*:* *M* is fully connected with q=1. Recall the block matrix configurations of the CM-model for the case $n_1=n_2=n/2$, and

$$A = I(1-c) + C^* - M^* - B \;=\; I(1-c) + C^* - M$$

where B is an nxn symmetric (off-diagonal block) random matrix with zero mean and variance $Var(b_{ij}) = \frac{m^2v^2}{3} = \sigma^2$. Adapting the work of Benaych-Georges & Nadakuditi (2011), and assuming q=1, the extreme eigenvalue (smallest) of A:

$$\lambda_1(A) \approx \begin{cases} 1 - c + \frac{n}{2}(c - m) & if \;\; m - c < \frac{\sqrt{2n}\sigma}{n/2}, \quad LH \;\; Border \\ 1 - c - \sqrt{2n}\sigma & otherwise, \quad RH \; Border \end{cases} \tag{2.5}$$

Where we recognize $\Lambda_2(M) = \sqrt{2n}\sigma$ is the second largest or subdominant eigenvalue of $M = M^* + B$.

We recognize the smallest eigenvalue from the uniform model is $\lambda_{1u} = 1 - c + \frac{n}{2}(c - m)$.

The above eigenvalue characterization Eqn.2.5 is tested numerically with examples in <u>**secion SN2-F.**</u>

The stability boundaries of the CM-model are found by setting $\lambda_1(A) = 0$. The two cases in eqn.2.5 lead to the two rays, the LH and RH borders, that generate the "inverted V-shaped" stability region (green&grey) in figure 2 main text and Fig. SN2-7 below.

Case 2. Connectance q<1.

It is assumed that a random proportion (1-q) elements $m_{ij} = m + b_{ij}$ of the matrix ***M*** are set to zero, so that the matrix has connectance q. The extreme eigenvalue (smallest) of A is:

$$\lambda_1 \approx \begin{cases} 1 - c + \frac{n}{2}(c - qm) & \text{if } qm - c < \frac{\sqrt{2n}\sigma}{n/2} \\ 1 - c - \Lambda_2(M) & \text{otherwise} \end{cases} \quad (2.6)$$

where one finds: $\Lambda_2(M) = \sqrt{2n}\sigma$ and $\sigma^2 = q(1-q)m^2 + \frac{qm^2v^2}{3}$. (2.7)

The first component $q(1-q)m^2$ represents the variance of elements M that have baseline value m while the second component represents the additional variance due to the structural disturbances b_{ij}

The stability boundaries of the CM-model are found by setting $\lambda_1(A) = 0$. The two cases in eqn.2.6 lead to the two rays, the LH and RH borders, that generate the "inverted V-shaped" triangle of stability stability (green&grey) in the figure below (Supplementary Figure 12):

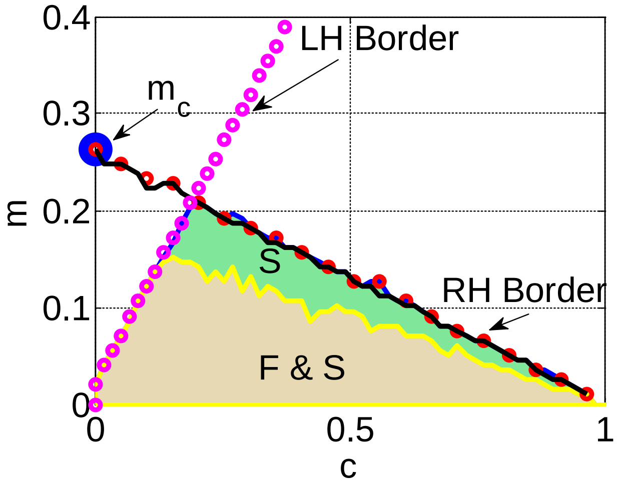

Supplementary Figure 12 **RIGHT HAND BOUNDARY** (red circles) occurs where $\Lambda_2(M)$ =1-c, where here $\Lambda_2(M)$ is the second largest or subdominant eigenvalue of *M*.

The RH boundary occurs when $\lambda_1(A) = 1 - c - \sqrt{2n}\sigma \quad = 0$

where $\sigma^2 = q(1-q)m^2 + \frac{qm^2v^2}{3}$.

Let the critical value m_c be the value of m when c=0. In the above plot m_c =0.27. We use simulations to compute m_c as a function of q, for different values of v and different sized communities n (red curves). It can also be computed from the above formula and should scale as $1/\sqrt{q(1-q)}$. Each simulation is an analysis of a random CM-matrix for a fixed set of parameter values, and then repeated so as to cover a wide range of parameter values. We then compare the stability analysis of the random matrices (red) to values with predictions (green curves in Supplementary Figure 13).

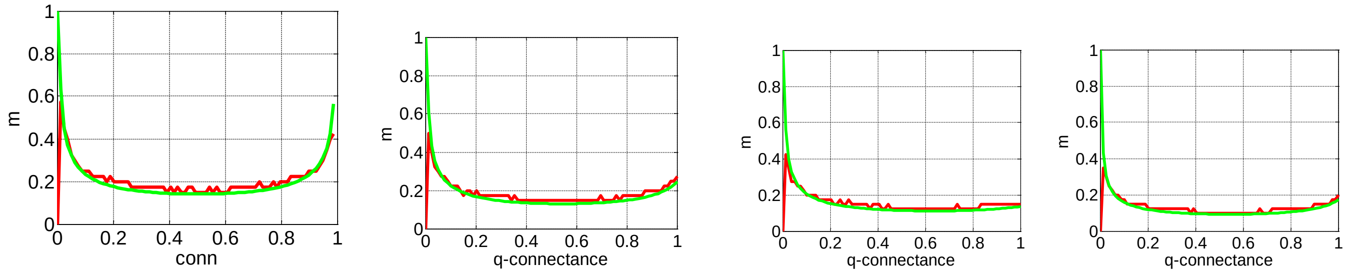

n=100, v=0.1 **v=0.5** **v=0.9** **n=200 v=0.5**

Supplementary Figure 13 red = simulated values. green is predicted value

Note that except for extreme values of q, connectance has little impact on $\boldsymbol{m_c}$. This is because the function $y = 1/\sqrt{q(1-q)}$ is essentially flat for 0.15<q<0.85 as shown in **Supplementary Figure 14.**

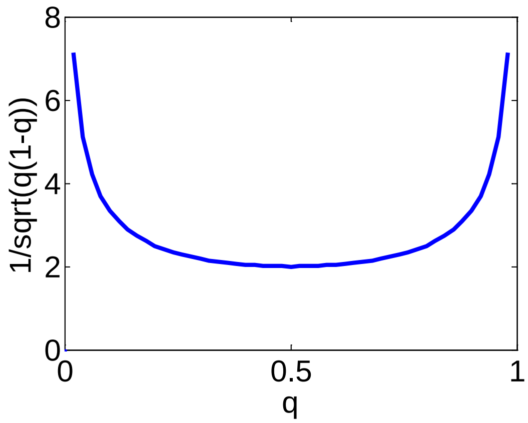

Supplementary Figure 14 $\boldsymbol{y = 1/\sqrt{q(1-q)}}$

In the examples below the validity of (Eqns.2.6&2.7) has been extensively tested out for the CM-model in detail. Note that the parameter space for which the matrix is stable is just the area under the triangle. The height of $\boldsymbol{m_c}$ $(\boldsymbol{red\ curve})$ is a good first approximation or proxy for the area of the Triangle of Stability which is shown in blue in Supplementary Fig.15 below.

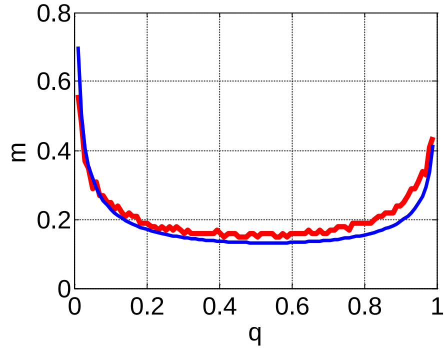

Supplementary Figure 15. n=100, v=0 Area of triangle of stability in blue; critical m_c in red

LH Border: Stability conditions for the uniform model:

In general, since the structurally disturbed CM-model can be considered as a perturbation from the uniform model, stability of the ensemble model requires that the uniform model be stable. We are interested in determining conditions for local stability of $A = I(1-c) + C^* - M^*$, where here the perturbation matrix B=0. The matrix M^* has elements of zeroes ($m_{ij} = 0$ when the network indicates no interaction) or the constant m ($m_{ij} = m > 0$, when there is a mutualistic interaction between species-i and –j.

As before, q is the connectance of matrix $\boldsymbol{M}$. Elementary consideration show that when $n_1=n_2=n/2$ and q=100% the smallest eigenvalue of A is:

$$\lambda_{1u} = 1 - c + \frac{n}{2}(c - qm) > 0$$

where stability requires this eigenvalue to be positive. Equivalently, stability requires

$$qm < \frac{2}{n} + \frac{(n-2)c}{n} \cong c \quad \text{for large } n, \quad \text{i.e.,} \quad q\,m < c$$

Example when $n_1=n_2$, q=1: Consider the CM-model for n=100, $n_1 = n_2$=50, m=0.3, q=1. The Feasible and stable systems as found numerically are plotted in grey in Figure S2-10, while the remaining area where the interaction matrix is stable in green.

Figure S2-10 shows that the LH border of stability is given by c ≅ m=0.3 , as predicted above. This corresponds to where the uniform model loses stability.

For the stochastic ensemble CM model, we have $\sigma = \frac{mv}{\sqrt{3}}$. Since q=1, the RH border is given by line $1 - c - \sqrt{2n}\sigma = 0$, or equivalently $v = \frac{\sqrt{3}(1-c)}{m\sqrt{2n}}$. For n=100, $n_1 = n_2$=50, m=0.3 this leads to a v-intercept of v=0.41 (when c=0) as observed in Supplementary Figure 16.

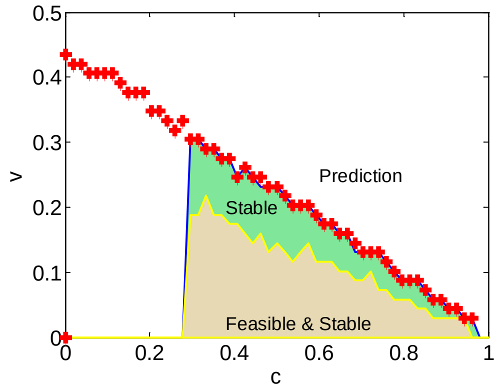

Supplementary Figure 16 CM-model $n_1 = n_2$=50, m=0.3 plot off RH-stability border where $\lambda_1 = 0$ (red +) on axes v versus c.

When $n_1 \neq n_2$.

More generally, assume there are n_1 animals and n_2 plants. Suppose the interaction matrix **A** has block competition interaction strengths set to c and all-to-all mutualistic interactions set with strength m. Animal species have identical characteristics so that each animal species-i has the same abundance A^*. Similarly plant species have the same abundance P^*. Eqn.7 (main text) at equilibrium then reads

$$A^*[1+(n_1-1)c] \quad - \quad P^* n_2 m \quad = 1$$

$$-A^* n_1 m \quad + \quad P^*[1+(n_2-1)c] = 1$$

Or $G\begin{bmatrix} A^* \\ P^* \end{bmatrix} = \begin{bmatrix} 1 \\ 1 \end{bmatrix}$ where the 2x2 matrix $G = \begin{bmatrix} 1+(n_1-1)c & -n_2 m \\ -n_1 m & 1+(n_2-1)c \end{bmatrix}$

The eigenvalues of G are:

$$\lambda = \frac{2+(n-2)c}{2} \pm \frac{\sqrt{(n_2-n_1)^2c^2+4n_1n_2m^2}}{2}$$

These two eigenvalues are also eigenvalues of the interaction matrix **A**, whose remaining (n-2) eigenvalues are all (1-c). A calculation shows $\lambda = 0$ when

$$m^2 = \frac{[1+(n_1-1)c]*[1+(n_2-1)c]}{n_1 n_2}, \qquad \text{(SN2.8)}$$

Or for large systems $m^2 \cong \frac{1}{n_2 n_1} + c^2$ i.e., $m \cong c$.

For systems with connectance q<1, the stability criterion is approximated as

$$(mq)^2 \cong \frac{1}{n_2 n_1} + c^2$$

EXAMPLE: ***$n_1 > n_2$*.,** **q=1:** CM-model n_1=102, n_2=12 Connectivity=q=1 v=0.5.

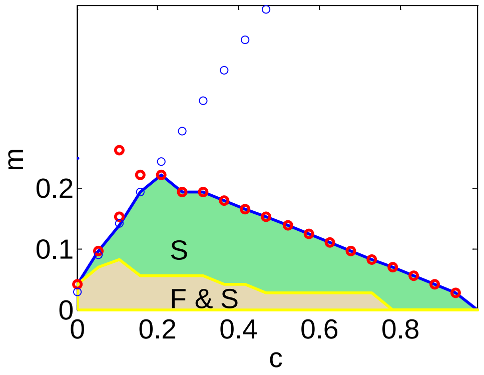

Supplementary Figure 17. The blue circles (Eqn. SN2.8) indicate parameters in which the uniform model loses stability, i.e., the LH border of stability, and is a plot of the curve: $m^2 = \frac{[1+(n_1-1)c]*[1+(n_2-1)c]}{n_1 n_2}$. We also use the above exact equation (SN2.8) for m^2 to plot the magenta circles in Fig.3 main text.

Alternatively the m-intercept m_{int} may be approximated as the value of m such that $\lambda(I+M) = 0$, since c=0, so that the LH border may be approximated as: $\boldsymbol{m = m_{int} + c/q}$

EXAMPLE: q<1. Estimating the LH stability border (red circles below) in CM model for lower connectance graphs q<1

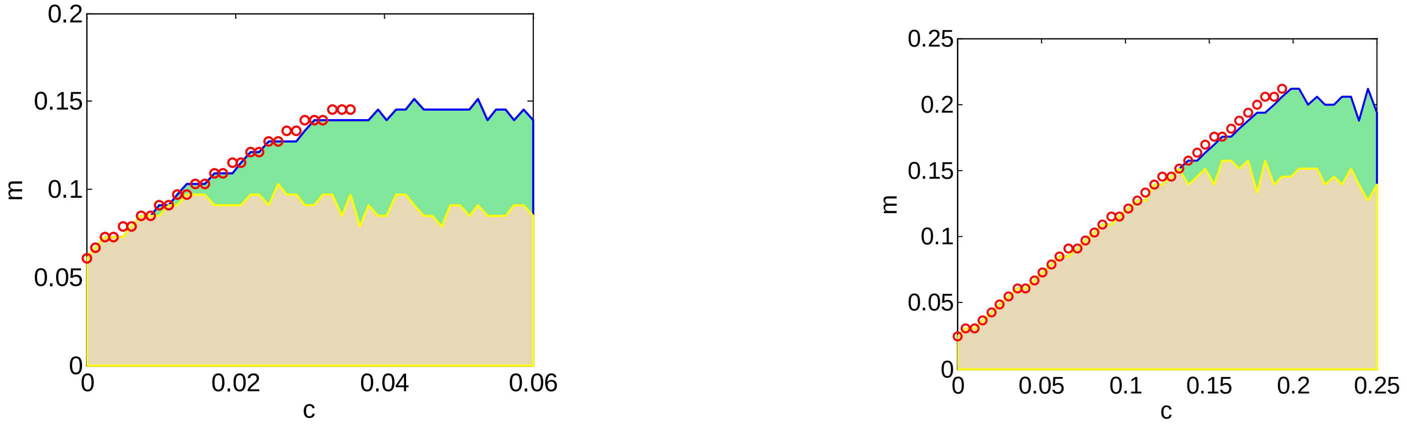

Figure Supplementary Figure 18. $n = 100$; $n_1 = n_2 = 50$,, Connectance ***q*=0.33** Connectance q =1

LH panel: For $n_1 = n_2$, $\lambda_{1u} = 0$ when $mq \cong \frac{2}{n} + c$

Expected intercept is $\boldsymbol{m_{int}}q$=2/n i.e., $\boldsymbol{m_{int}}$=0.06. The expected slope should corresponds to slope =1/q=3 (m=qc). The slope in Supplementary Figure 18a is approximately 3.

RH panel: *q*=1, Expected intercept is $\boldsymbol{m_{int}}$=1/50=0.02.

The slope should correspond *c=mq*, or *m=1c*. The slope in the figure is approximately 1

2E. All feasible CM-systems are stable. They are in fact D-Stable

The goal of this section is to show that:

Feasibility of the CM-system is lost before stability of the interaction matrix A.

We suppose that the CM-model is stable and a destabilizing parameter such as $\rho(\boldsymbol{M})$ or possibly c or σ is gradually increased. For the first population, for example, at equilibrium the equations are of the form:

$$(1-c)N_1^* + c\sum_1^{n_1} N_i^* \; - \sum_{j=n_2+1}^{n} m_{1j} N_j^* = 1 \qquad (2.4)$$

where $m_{ij} \geq 0$. Two scenarios are observed:

Scenario I) This scenario corresponds to instability due to the RH-ray of the Triangle of Stability described in SN2-D with population blowup seen in the figure below (SN2-13). Referring to the analysis for competition systems (Section SN1-H) it was shown that as $|A| \to 0$, at least one equilibrium population must blow up, (eg $N_1^* \to \infty$). This also applies to the CM-model -- as also seen in simulations of Fig.SN2-13.

Conditions that lead to $|A| \to 0$, derive from the critical eigenvalue which for the RH-ray of the triangle corresponds to: λ =1-c- $\Lambda_2(M)$.

Recall that by Taussky's theorem SN2-1 the eigenvalue $\Lambda_2(M) \in \mathbb{R}$, must be a real number. Therefore loss of stability will always translate into $|A| = 0$ (on the RH border) and thus population blowup as explained in SN1-H for the competition equations.

One possibility for Eqn.2.4 to be consistent with this, is that at least another equilibrium population N_k^* ($k \leq n_1$) must simultaneously decrease and explode negatively downwards $N_k^* \to -\infty$. (See Fig.SN2-13a). In this case, as for the competition equations, feasibility of the system must be lost before stability of the interaction matrix $\boldsymbol{A}$.

As for the competition equations, when $\rho(B') < 1$, we can expand the inverse matrix A to obtain the first order approximation of the population of the i'th species at equilibrium: $N_i^* \approx \kappa(1 - \sum_{j=1}^{n} b'_{ij})$

Recall that the b_{ij} are random perturbations having mean zero and variance $\mathrm{Var(b_{ij})} = \sigma^2$. The populations N_i^* are distributed normally with mean κ and spread out symmetrically in an envelope about κ. Hence as σ increases, the window increases, and roughly 50% of the populations increase while 50% decrease similar to Supplementary Figure 19a.

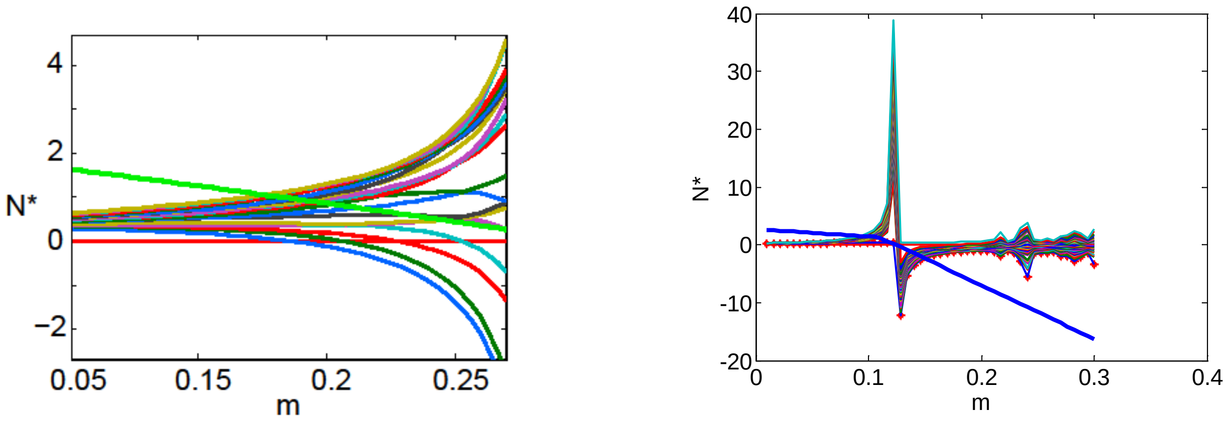

a) **b)**

Supplementary Figure 19. **a**) Scenario I. Equilibrium abundances of CM-model for *n=20* species ($n_1 = n_2 = 10; c = 0.2,\ v = 0.4,\ q = 0.7$) plotted versus interaction strength *m*. Feasibility is lost at *m=0.18.* Critical eigenvalue of interaction matrix *A* (green line) zeroes at the "blow-up" point $m = 0.28$ where $|\boldsymbol{A}| = \boldsymbol{0}$ and stability is lost.
b) Scenario II $n_1 = n_2 = n/2$=30, c=0.1, v=0.7, q=1 Blow up occurs close to when $m \simeq c$. The minimum eigenvalue,here is real and plotted in blue as function of *m*. All population levels N_i^* plotted as a function of *m*.

Scenario II) The second scenario depends on the uniform model and corresponds to the LH-arm of the Triangle of Stability. Recall that the stochastic ensemble model is a perturbation of the uniform model. There is a parameter range for which the uniform model is unstable – namely on the LH-edge of the Triangle of Stability (see Section SN2-D). When the uniform model is on the brink of stability its dynamics appear as in Fig. SN2-13b&14b. All *n*-equilibrium populations increase or "blow up" as *m* increases until stability is lost when *m*=0.24 (LH panel). After this, the populations then all suddenly jump to large negative numbers and feasibility is suddenly lost. The behaviour can also be seen to be consistent with Eqn.2.4. The behaviour of the uniform model (Fig. SN2-14 LH) is easy to derive analytically (following analysis formulated in SN2-D).

For parameters close to this unstable parameter regime, the stochastic ensemble model essentially mimics this same behaviour as shown in Supplementary Figure 20 RH panel with v=0.4. As expected, when equilibrium numbers attain large values, the effects of noise perturbations are negligible. We will see that the eigenvalue associated with the uniform model is real (we refer to it as the Perron eigenvalue – see SN2-C), and

thus we know that $|\boldsymbol{A}|$ passes through zero ($|\boldsymbol{A}| = \mathbf{0}$) when populations flip to huge negative values, and both feasibility and stability are lost together. (This scenario corresponds to the LH-ray of the Triangle of Stability described in SN2-D.)

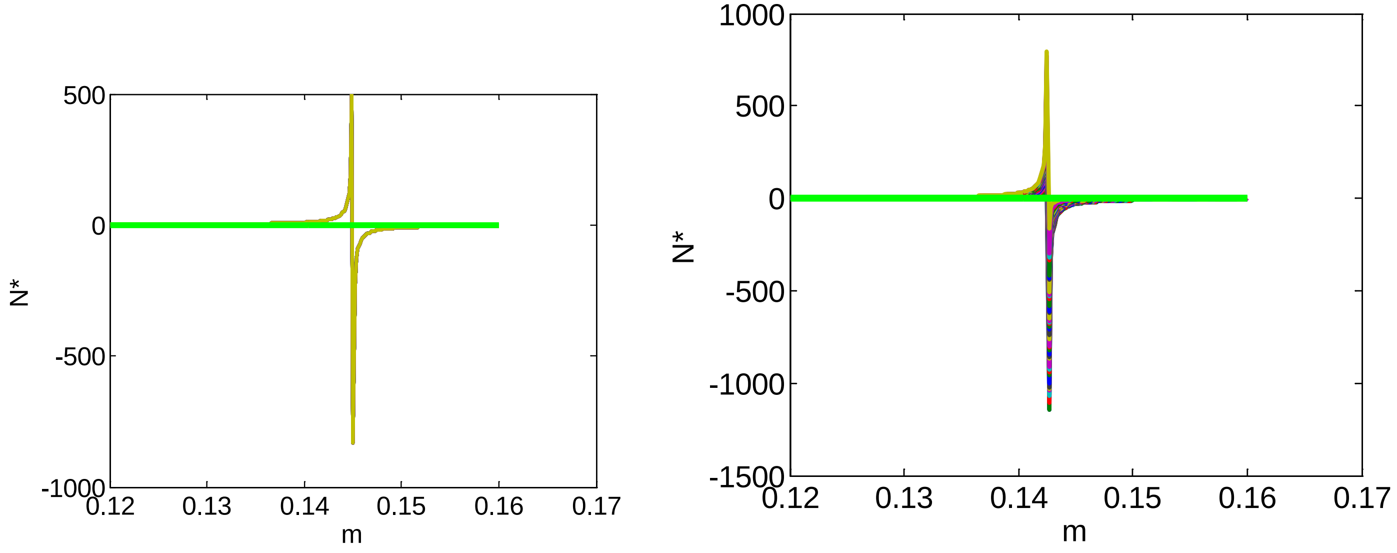

Supplementary Figure 20. LH panel CM-model **uniform model** v=0 (**B**=0), with $n_1 = n_2 = 20; c = 0.1$.**RH panel**. Stochastic CM-model where uniform model is close to instability. As a) but with v=0.8 and thus perturbations $\boldsymbol{B} \neq 0$

In summary:

1) For the CM-model, feasibility of the system is lost at or (more typically) before stability of the interaction matrix A.
2) RSB have, through exhaustive numerical simulations, concluded that feasible CM-models are D-stable. Thus a locally stable interaction matrix A implies that the stability matrix S=DA is locally stable.
3) As such, we expect all feasible CM-systems to be locally stable as shown in **Supplementary Figure 21** (for n=14). Results found there are deliberately for small *n*, which is where exceptions to the claim might be expected.
4) Stability is lost by the critical eigenvalue, which is real, changing sign from positive to negative and passing through zero. Unlike the competition equations, this translates into attaining $|\boldsymbol{A}| = \mathbf{0}$, and thus instability can always be associated with population blowup.
5) Because stability is lost by a real eigenvalue of A changing sign from positive to negative, we can be sure from our earlier discussions (eg SN1-I) that A is D-stable. (See also Johnson 1974).

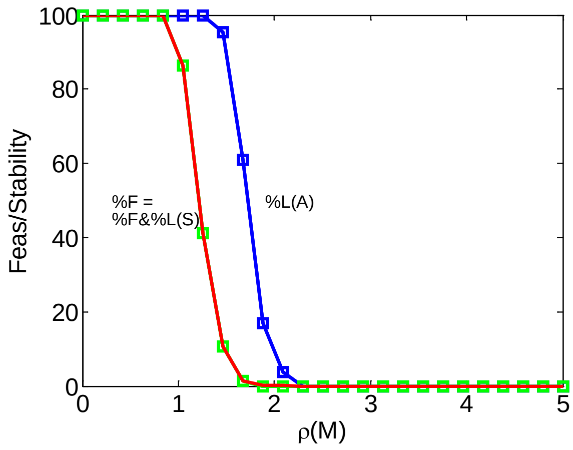

Supplementary Figure 21. Percentage of 500 CM-systems ($n_1 = n_2 = 7; c = 0.2; q = 0.7$) that are feasible (red) as a function of $\rho(M)$, and percentage of feasible systems that are locally stable (green square). These two curves are completely identical. Percentage of interaction matrices A that are locally stable (blue line) and globally stable (blue square) .

2F Randomized Networks and Nestedness

There have been strong arguments suggesting the widely observed nestedness arrangement of species in empirical networks has a major impact on structural stability *(6,7*). Yet after randomizing the links *(25,26)* in the CSF matrix, the feasibility and stability characteristics are not perceptibly different as seen from comparing Supplementary Figure 22 LH with its randomized counterpart in the RH panel. Another example may be seen in Figure 23.

The randomization procedure randomizes the location of all links in the network via a switching procedure, subject to the constraint that the in-degree and out-degree of each node remains fixed (*Stone and Roberts 1990*). The network's degree distribution controls the eigenvalues of ***M*** *(Feng and Takemoto 2014)*, and has a close relationship to nestedness. As such, realistic randomizations that maintain degree distribution have little overall impact, given eigenvalues change little. While nestedness is known to affect the eigenvalue distribution of M (Staniczenco et al. 2013), the impact is marginal compared to other parameters of our model (eg., n_1,n_2, c,m,v,q). Fig.3 main text illustrates these points further.

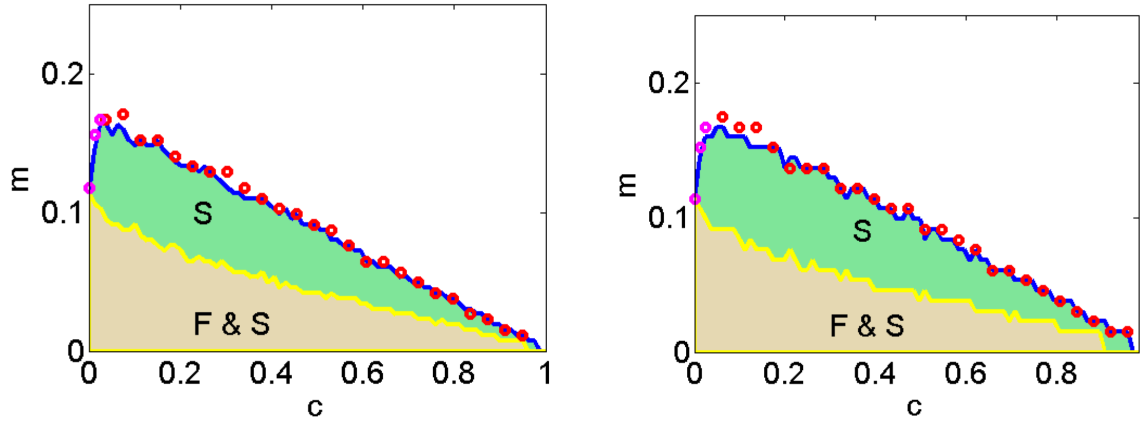

Canadian fir-forest randomized network

Supplementary Figure 22. Feasibility and stability properties of Canadian fir-forest matrux (from 24)

Files from RSB used in Fig.3 main text: M_PL_004,006,13,17,19,24,25,33 M_SD_001-6,8-12,14,20,23

Supplementary Note 3: Analysis of empirical pollination matrices

The Mutualistic Benefit case of δ=1

Here we first test whether the "second" eigenvalue criterion (Eq.1o main text) holds for mutualistic benefit of δ=1

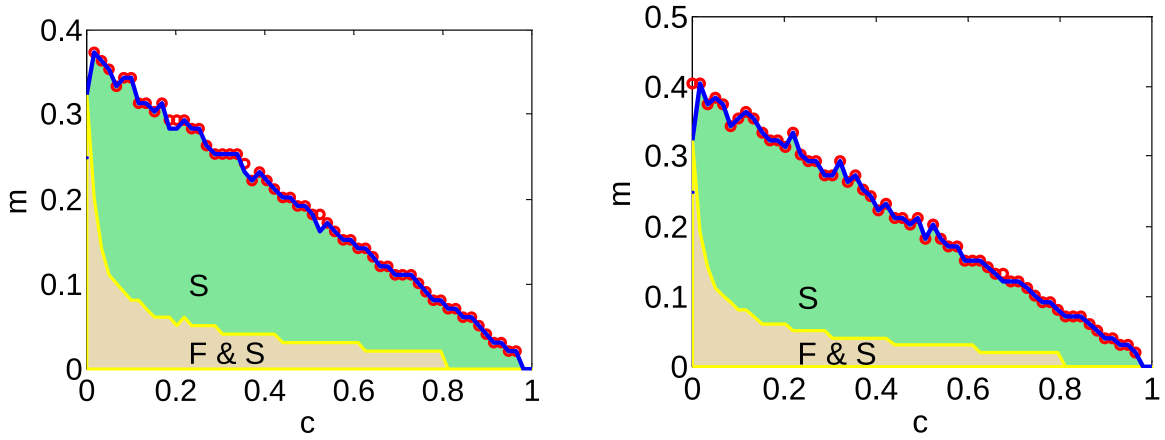

Supplementary Figure 23. v=0.5, δ=1, Real Data Randomized Data δ=1

Recall that RSB define mutualistic interactions as: $m_{ij} = (m_{ij}^{0} y_{ij})/k_i^{\delta}$

where $y_{ij} = 1$ if species-i and -j interact and zero otherwise. k_i is the number of mutualistic interactions of species-i, m_{ij}^{0} represents the level of mutualistic strength. The figures in the main text make use of mutualistic benefit δ=0.

Here we use δ=1 and reanalyse the data from matrix as used in Fig.3c (main text;, the Canadian forests). The above LH figure (Supplementary Figure 23) shows the 2nd eigenvalue criterion Eqn.10 (red circles) matches very closely to true stability of the full interaction matrix A.

The graph on the right also shows that randomizing the data while maintaining the network's degree distribution has little impact on the analysis.

Returning to δ=0

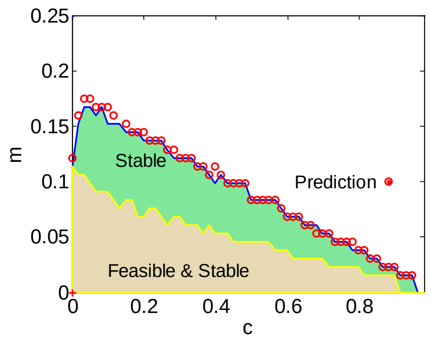

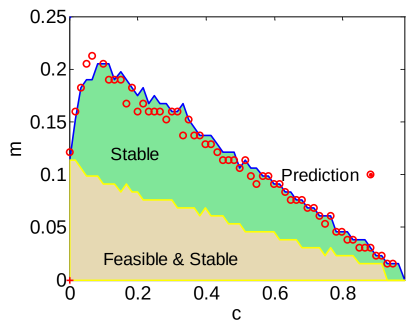

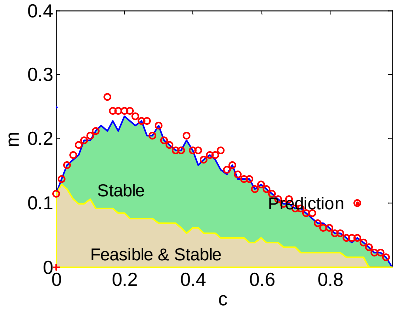

PL_004 m=0.03 q=13%   PL_006 q=14%   SD_002 q=43%

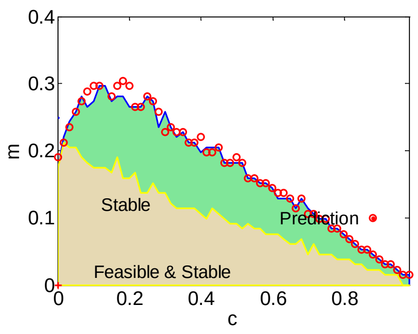

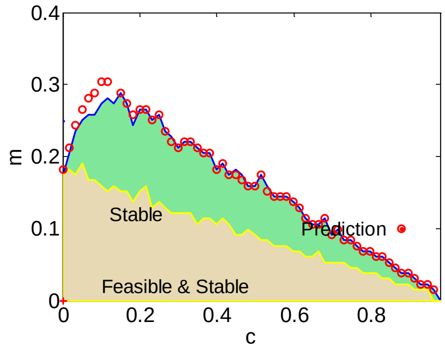

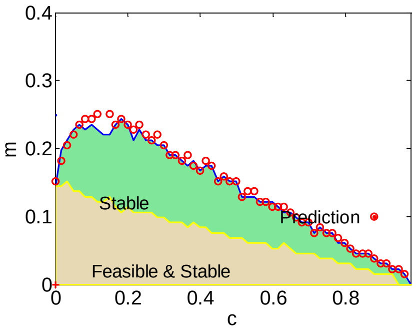

SD_001 q=35%   SD_003 q=17%   SD_004

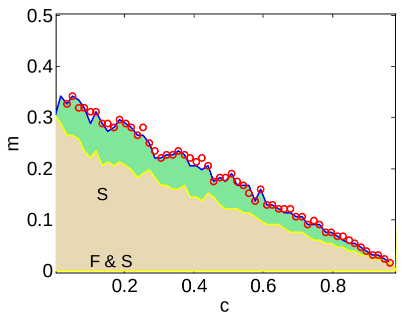

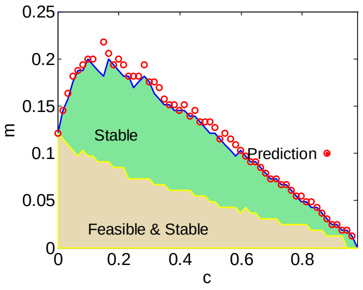

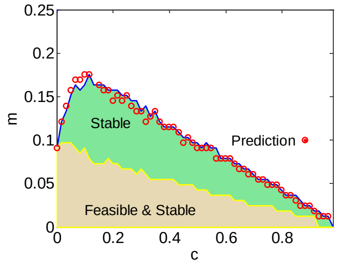

SD 005 q=14%   M_PL_033 Ottawa Canada   Rainforest Trinidad, M_SD_010

Animals=34 Plants=13
Connectance q=32%

13 animals=34, plants=50, q=34%

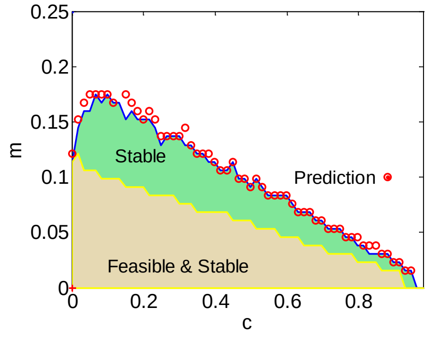

M_PL_019

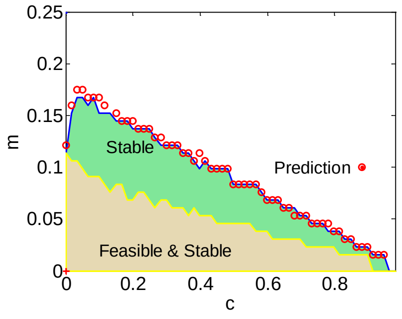

M_PL_004; q=17%

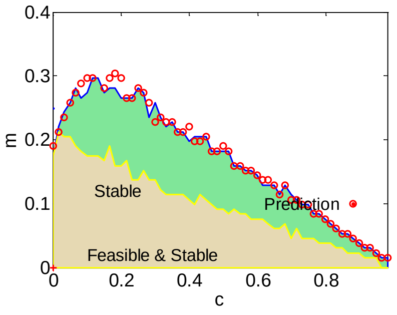

SD_001

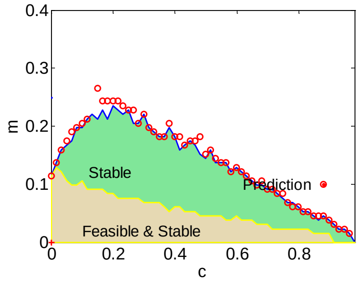

M_SD_002

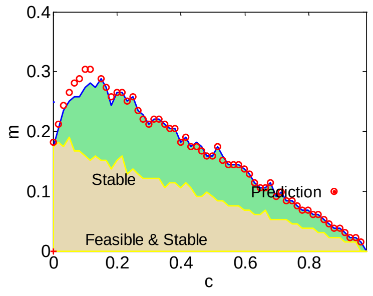

M_SD_003

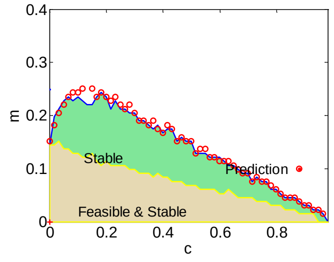

M_SD_004

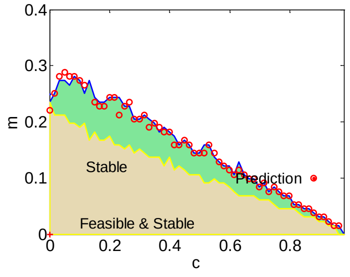

M_SD_006

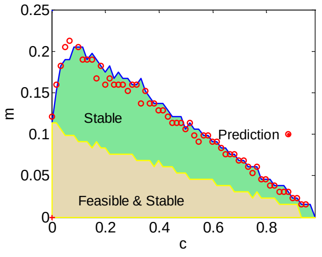

M_PL_006

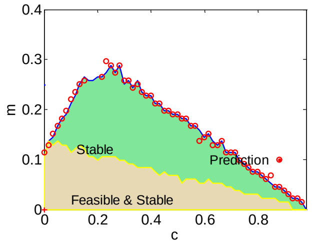

M_SD_008 q=0.69

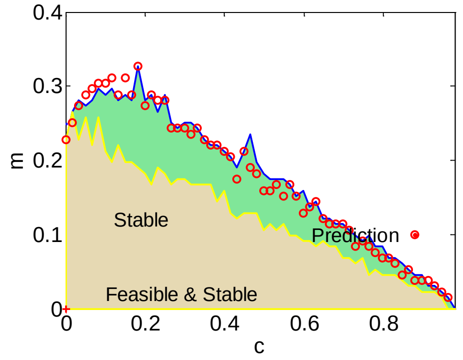

M_SD_009 n1=7 n2=18 CONN=31%

Supplementary Figure 24. We analyse 21 real empirical mutualism networks as analysed by RSB and references therein. (Data available at www.web-of-life.es) **Red circles** indicate stability border determined by 2nd eigenvalue criterion Eq.6 main text.

Supplementary Note 4: The Google Matrix

Constructing an adjacency matrix P of links for the entire world wide web would require more than 25 billion rows and columns and would need to quantify the hyperlinks between all documented webpages. The form of the final Google matrix chosen by Brin and Page in their PageRank algorithm (Brin&Page 1998) is $G = P + C$. Here P is the scaled row-stochastic matrix of hyperlinks between web pages. and C a matrix of low-rank (specifically all entries ones (or other identical constant), just as in the competition model). The massive size of these matrices implies that computing eigenvalues/vectors requires extensive time and huge computing resources to deal with the many billions of multiplications needed, if only to multiply the Google matrix G just once.

Because the maths shows that it is possible to almost ignore the rank-one perturbation C for some specific applications, Brin and Page (1999) understood that they only had to work computationally with the hyperlink matrix P, which is a much easier prospect In 2004 the estimated number of outlinks for a webpage was 52. Thus for a typical row of the hyperlink matrix only 52 of the 25 billion elements are nonzero, so the majority of elements of P are zero i.e., P is very sparse. Thus simple iterative methods can solve the PageRank algorithm by working with the matrix P to find eigenvectors/values of the Google matrix G. This takes a matter of hours for the whole world wide web. The algorithm is considered one of the best page ranking systems available.

The same Google matrix concept is described in the thesis of Stone (1988), but in the context of biological modeling, making it possibly the first application of the Google matrix some ten years before its invention by Google (Brin and Page (1998)). Both Stone (1988) and Brin and Page (1998) faced the difficulty of solving large network systems having numerous pairwise interactions. Both worked with properties of the Google matrix, because its special structure allows interactions of low rank (eg the matrix C) to be effectively discarded from the calculation.

The paper of Brin and Page' (1998) has since inspired a surge of research activity into the mathematics of their Google matrix and its variants (eg. Haveliwala (1999), Ding and Zhou (2007), Wills (2006), Horn and Serra-Capizzano (2008), Cicone and Serra-Capizzano (2010), Zhou (2011)). It was recently pointed out that the eigenvalue property was also noted by the mathematician Brauer (1952) in a pure mathematics journal, where it went largely unnoticed for decades. The property was discovered independently by Stone *(1988)* who applied it to study the stability of large complex biological systems. One of Stone's (1988) main contributions was to show that for competition systems based on nonlinear differential equations such as the Lotka-Volterra model, the important stability matrix **S** has the eigenvalue properties of the Google matrix. This assumes there is a positive (feasible) equilibrium. Further work is needed to generalize these results to other structured systems, such as the CM-system of the present paper using the Google matrix approach.

Additional References

1. Allesina, S. & Tang, S. The stability–complexity relationship at age 40: a random matrix perspective. *Population Ecology*, *57*, 63-75 (2015a). .

2. Berman, A. & Grone, R. Bipartite completely positive matrices. In *Mathematical Proceedings of the Cambridge Philosophical Society* **103**, 269-276 (1988).

3. Berman, A. & R.J. Plemmons, R.J.. Nonnegative matrices. *The Mathematical Sciences, Classics in Applied Mathematics*, *9*.   (1979).

4. Cicone A., Serra-Capizzano S. Google PageRanking problem: The model and the analysis. Jnl. Of Computational and Applied Mathematics 234, 3140-3169 (2010).

5. Furedi, Z. & Komlos, J. The eigenvalues of random symmetric matrices. *Combinatorica* 1, 233–241. (1981).

6. Geman, S. A limit theorem for the norm of random matrices. *The Annals of Probability* 252-261 (1980).

7. Gilpin, M.E. & Case, T.J. Diffuse multispecies competition: sequence dependent multiple domains and domino collapse. In: Differential Equations and Applications in Ecology, Epidemics, and Population Problems (Freedman, W., ed.) New York: Academic Press (1981).

8. Horn, R.A.H. & Serra-Capizzano, A. A general setting for the parametric google matrix. *Internet Mathematica* 3(4):385-411 (2008).

9. Johnson, C.R. Sufficient conditions for D-stability. *Journal of Economic Theory* 9, 53-62. (1974).

10. Kokkoris, G.D., Jansen, V.A.A., Loreau, M. & Troumbis, A.Y. Variability in interaction strength and implications for biodiversity. *Journal of Animal Ecology* 71, 362-371. (2002).
11. Lidskii, V.B. Perturbation theory of non-conjugate operators."*USSR Computational Mathematics and Mathematical Physics* 6, 73-85. (1966).

12. May, R.M. Theoretical ecology. Principles and applications. (1976).

13. Mehl, C., Mehrmann, V, A.C.M. Ran, A.C.M. & Rodman, L. Eigenvalue perturbation theory of classes of structured matrices under generic structured rank one perturbations. *Linear Algebra and its Applications* 435, 687-716 (2011).

14. Moro, J., Burke, J.V. & Overton, M.L.. On the Lidskii--Vishik--Lyusternik Perturbation Theory for Eigenvalues of Matrices with Arbitrary Jordan Structure. *SIAM Journal on Matrix Analysis and Applications* 18, 793-817 (1997).

15. Plemmons, R.J. M-matrix characterizations. I—nonsingular M-matrices. *Linear Algebra and its Applications*, *18*, 175-188 (1977).

16. Pomerantz, M.J. & Gilpin, M.E. Community covariance and coexistence. *Journal of Theoretical Biology* 79, 67-81 (1979).

17. Ran, A.C.M. & Wojtylak, M.. Eigenvalues of rank one perturbations of unstructured matrices. *Linear Algebra and its Applications* 437, 589-600. (2012).

18. Roberts, A. When will a large complex system be viable? Environmental Discussion Paper, Graduate School of Environmental Science, Monash University, Melbourne, Australia. (1989).

19. Staniczenko, P.P.A., Kopp, J.C., & Allesina, S. The ghost of nestedness in ecological networks. *Nature Communications* **4**, 1391 (2013)

20. Stone, L. & Roberts, A. Conditions for a species to gain advantage from the presence of competitors. *Ecology*, 1964-1972 (1991).

21. Stone, L. & Roberts, A. The checkerboard score and species distribution. *Oecologia* **85**, 74-79 (1990).

22. Tao, T. Outliers in the spectrum of IID matrices with bounded perturbations.

23. Taussky, O. Positive-definite matrices and their role in the study of the characteristic roots of general matrices. *Advances in Mathematics*, *2*, 175-186. (1968).

24. Wigner, E.P. Random matrices in physics. *SIAM Review* 9:1-23. (1967).

25. Zhou, Y. On the eigenvalues of specially low-rank perturbed matrices. *Applied Mathematics and Computations*. 217, 10267-10270. (2011).

26. Wills, R.S. Google's PageRank: The math behind the search engine. *The Mathematical Intelligencer* **28**, 1677-1696. (2006).

27. Zhou Y. On the eigenvalues of specially low-rank perturbed matrices. Applied Mathematics and Computation. 217, 10267-10270 (2011).

28. Brauer, A. Limits for the characteristic roots of a matrix IV: Applications to stochastic matrices, Duke Math J. **19**, 75-91 (1952).
